# Identification of *Shigella flexneri* IcsA Residues Affecting Interaction with N-WASP, and Evidence for IcsA-IcsA Co-Operative Interaction

**DOI:** 10.1371/journal.pone.0055152

**Published:** 2013-02-06

**Authors:** Min Yan Teh, Renato Morona

**Affiliations:** Discipline of Microbiology and Immunology, School of Molecular and Biomedical Science, University of Adelaide, Adelaide, South Australia, Australia; Monash University, Australia

## Abstract

The *Shigella flexneri* IcsA (VirG) protein is a polarly distributed outer membrane protein that is a fundamental virulence factor which interacts with neural Wiskott-Aldrich syndrome protein (N-WASP). The activated N-WASP then activates the Arp2/3 complex which initiates *de novo* actin nucleation and polymerisation to form F-actin comet tails and allows bacterial cell-to-cell spreading. In a previous study, IcsA was found to have three N-WASP interacting regions (IRs): IR I (aa 185–312), IR II (aa 330–382) and IR III (aa 508–730). The aim of this study was to more clearly define N-WASP interacting regions II and III by site-directed mutagenesis of specific amino acids. Mutant IcsA proteins were expressed in both smooth lipopolysaccharide (S-LPS) and rough LPS (R-LPS) *S. flexneri* strains and characterised for IcsA production level, N-WASP recruitment and F-actin comet tail formation. We have successfully identified new amino acids involved in N-WASP recruitment within different N-WASP interacting regions, and report for the first time using co-expression of mutant IcsA proteins, that N-WASP activation involves interactions with different regions on different IcsA molecules as shown by Arp3 recruitment. In addition, our findings suggest that autochaperone (AC) mutant protein production was not rescued by another AC region provided *in trans*, differing to that reported for two other autotransporters, PrtS and BrkA autotransporters.

## Introduction

*Shigella flexneri* is a human pathogen that causes bacillary dysentery by infecting and colonising the colonic epithelium [1]. IcsA (VirG), one of the key virulence factors of *S. flexneri*, is polarly distributed at the outer membrane (OM). IcsA is required for inter- and intracellular spreading of *S. flexneri* within the host intestinal epithelium [2], [3], [4], [5], [6]. Inside the host cytoplasm, *Shigella* multiplies and interacts with the host actin regulatory protein neural Wiskott-Aldrich syndrome protein (N-WASP), which in turn activates the Arp2/3 complex that initiates actin polymerisation by recruiting the globular actin monomers to form the filamentous actin (F-actin) comet tails [7], [8], [9]. The formation of F-actin comet tails allows bacterial actin-based motility (ABM) [2], [7], [8].

IcsA is a member of the autotransporter (AT) family (Type Va secretion system), the largest family of secreted proteins in Gram-negative bacteria [10], [11]. Like other family members, IcsA consists of three major domains: an extended N-terminal signal sequence (amino acids [aa] 1–52), a functional passenger α-domain (aa 53–758), and a C-terminal translocation β-domain (aa 759–1102) that mediates the translocation of the passenger domain across the OM via the BAM (beta-barrel assembly machine) complex [11], [12], [13], [14], [15]. The β-barrel domain is anchored in the OM, while the functional IcsA passenger domain is exposed on the bacterial surface and is responsible for N-WASP recruitment and ABM [3], [8].

In spite of the diversity in sequence, function and length, the passenger domains of most ATs possess a β-helical structure [16]. IcsA was recently classified to a subgroup of self associating ATs (SAATs) that mediate bacterial aggregation and biofilm formation [17], [18]. The ability of IcsA to self-associate has recently been shown via reciprocal co-precipitation of differentially epitope-tagged IcsA proteins [19]. A putative autochaperone (AC) region at the C-terminal of IcsA passenger domain (aa 634–735), which forms part of the SAAT domain, is required for IcsA biogenesis [18], [20]. To date, only the crystal structure of the IcsA AC region (aa 591–758) is available, and it possesses two coils of a right-handed parallel β-helix [21].

N-WASP is a key regulator of the actin cytoskeleton and functions as a link between signalling pathways and *de novo* actin polymerisation, leading to host cell motility and morphological changes [8], [22], [23], [24], [25]. IcsA_103–433_ which contains glycine-rich repeats (GRRs; aa 117–307) is sufficient for N-WASP binding *in vitro* [24], [26], and *in vitro* assays showed that IcsA_53–508_ is adequate to induce actin polymerisation [8]. Five amino acids (5 aa) linker-insertion mutations (IcsA_i_) [20] confirmed the involvement of aa 185–312 (N-WASP interacting region [IR] I), and a second region (aa 330–382 [N-WASP IR II]) in N-WASP recruitment. N-WASP IR II overlapped with the *S. flexneri* IcsB and host autophagy protein, Atg5, binding region (aa 320–433) [27]. In addition, a new IcsA region (aa 508–730 [N-WASP IR III]) that contains the IcsA AC region was also suggested to be important for N-WASP recruitment and F-actin comet tail formation [20]. However, specific N-WASP binding/interaction site or residues within IcsA have not yet been reported.

From the 5 aa linker-insertion mutagenesis study, IcsA_i_ mutants (IcsA_i633_, IcsA_i643_, IcsA_i677_, and IcsA_i716_) with mutational alterations within the AC region have markedly reduced protein production in *S. flexneri* strain with native LPS O-antigen (LPS Oag) (smooth LPS, S-LPS) and hence, were unable to form plaques, recruit N-WASP or form F-actin comet tail [20]. When the same IcsA_i_ mutants were expressed in *S. flexneri* strain without LPS Oag (rough LPS, R-LPS), IcsA_i_ mutant production was restored to a level comparable to wild-type (WT) IcsA [28]. With the restoration of IcsA_i_ production in *S. flexneri* R-LPS strains, we have identified that IcsA_i633_, IcsA_i643_, IcsA_i677_, but not IcsA_i716_, were able to recruit N-WASP and form F-actin comet tails [28].

In this study, we investigated the ability of IcsA mutants with point mutations within N-WASP IR II and III to recruit N-WASP and form F-actin comet tails in both S-LPS and R-LPS *S. flexneri* backgrounds. Using this approach, we have identified specific residues affecting N-WASP binding/interaction. In addition, we have also co-expressed IcsA mutant proteins with mutations at two N-WASP interaction regions, and showed for the first time that all three N-WASP interacting regions are essential for N-WASP and Arp3 recruitment, supporting a role for oligomeric IcsA in this interaction.

## Materials and Methods

### Ethics Statement

The anti-IcsA antiserum was produced under the National Health and Medical Research Council (NHMRC) Australian Code of Practice for the Care and Use of Animals for Scientific Purposes and was approved by the University of Adelaide Animal Ethics Committee.

### Bacterial Strains and Plasmids

The strains and plasmids used in this study are listed in Table 1.

**Table 1:** Bacterial strains and plasmids.

Strain or plasmid	Relevant characteristics^a^	Reference or source
***E. coli*** ** K-12**		
DH5α	Cloning host	Gibco-BRL
XL-10 Gold	Tc^R^ Δ(*mcrA*)*183* Δ(*mcrCB-hsdSMR-mrr*)*173 endA1 supE44 thi-1 recA1 gyrA96 relA1 lac*Hte [F *proAB lacI* ^q^ *Z*Δ*M15* Tn*10* (Tc^R^) Amy Cm^R^	Stratagene
***S. flexneri***		
2457T	*S. flexneri* 2a wild type	Laboratory collection
RMA2041	2457T Δ*icsA*::Tc^R^	[43]
RMA2043	RMA2041 Δ*rmlD*::Km^R^	[43]
MYRM329	RMA2041 [pMYRM315]	This study
MYRM331	RMA2041 [pMYRM317]	This study
MYRM332	RMA2041 [pMYRM318]	This study
MYRM333	RMA2041 [pMYRM319]	This study
MYRM336	RMA2041 [pMYRM322]	This study
MYRM338	RMA2041 [pMYRM324]	This study
MYRM346	RMA2041 [pMYRM340]	This study
MYRM352	RMA2041 [pMG55]	This study
MYRM353	RMA2043 [pMG55]	This study
MYRM354	RMA2043 [pMYRM315]	This study
MYRM355	RMA2043 [pMYRM317]	This study
MYRM356	RMA2043 [pMYRM318]	This study
MYRM357	RMA2043 [pMYRM319]	This study
MYRM359	RMA2043 [pMYRM322]	This study
MYRM360	RMA2043 [pMYRM324]	This study
MYRM362	RMA2043 [pMYRM340]	This study
MYRM562	RMA2041 [pMYRM536]	This study
MYRM563	RMA2041 [pMYRM537]	This study
MYRM564	RMA2041 [pMYRM538]	This study
MYRM565	RMA2041 [pMYRM539]	This study
MYRM567	RMA2041 [pMYRM543]	This study
MYRM575	RMA2041 [pMYRM556]	This study
MYRM568	RMA2041 [pMYRM544]	This study
MYRM569	RMA2041 [pMYRM545]	This study
MYRM570	RMA2041 [pMYRM546]	This study
MYRM571	RMA2041 [pMYRM547]	This study
MYRM572	RMA2041 [pMYRM548]	This study
MYRM577	RMA2041 [pMYRM549]	This study
MYRM596	RMA2041 [pMYRM588]	This study
MYRM598	RMA2041 [pMYRM590]	This study
MYRM600	RMA2041 [pMYRM592]	This study
MYRM602	RMA2041 [pMYRM595]	This study
MYRM604	RMA2043 [pMYRM592]	This study
MYRM606	RMA2043 [pMYRM595]	This study
MYRM610	RMA2041 [pMYRM608]	This study
MYRM612	RMA2043 [pMYRM608]	This study
MYRM646	RMA2041 [pSU23-IcsA]	This study
MYRM647	RMA2041 [pSU23-IcsA::BIO]	This study
MYRM648	RMA2041 [pSU23-IcsA_i248_]	This study
MYRM649	RMA2041 [pSU23-IcsA::BIO Y716G D717G]	This study
MYRM650	RMA2043 [pSU23-IcsA]	This study
MYRM651	RMA2043 [pSU23-IcsA::BIO]	This study
MYRM652	RMA2043 [pSU23-IcsA_i248_]	This study
MYRM653	RMA2043 [pSU23-IcsA::BIO Y716G D717G]	This study
MYRM685	RMA2041 [pSU23]	This study
MYRM655	RMA2043 [pSU23]	This study
MYRM656	RMA2041 [pSU23-IcsA_i248_]+[pIcsA::BIO Y716G D717G]	This study
MYRM657	RMA2041 [pSU23-IcsA_i248_]+[pIcsA::BIO V382R]	This study
MYRM658	RMA2041 [pSU23-IcsA::BIO Y716G D717G]+[pIcsA_i248_]	This study
MYRM659	RMA2041 [pSU23-IcsA::BIO Y716G D717G]+[pIcsA::BIO V382R]	This study
MYRM660	RMA2041 [pSU23-IcsA]+[pBR322]	This study
MYRM661	RMA2041 [pSU23-IcsA::BIO]+[pBR322]	This study
MYRM662	RMA2041 [pSU23-IcsA_i248_]+[pBR322]	This study
MYRM663	RMA2041 [pSU23-IcsA::BIO Y716G D717G]+[pBR322]	This study
MYRM669	RMA2041 [pSU23-IcsA]+[pIcsA_i248_]	This study
MYRM670	RMA2041 [pSU23-IcsA]+[pIcsA::BIO Y716G D717G]	This study
MYRM671	RMA2041 [pSU23-IcsA]+[pIcsA::BIO V382R]	This study
MYRM672	RMA2041 [pSU23-IcsA::BIO]+[pIcsA_i248_]	This study
MYRM673	RMA2041 [pSU23-IcsA::BIO]+[pIcsA::BIO Y716G D717G]	This study
MYRM674	RMA2041 [pSU23-IcsA::BIO] +[pIcsA::BIO V382R]	This study
MYRM686	RMA2041 [pSU23]+[pIcsA]	This study
MYRM687	RMA2041 [pSU23]+[pIcsA::BIO]	This study
MYRM688	RMA2041 [pSU23]+[pIcsA_i248_]	This study
MYRM689	RMA2041 [pSU23]+[pIcsA::BIO Y716G D717G]	This study
MYRM690	RMA2041 [pSU23] +[pIcsA::BIO V382R]	This study
MYRM691	RMA2043 [pSU23-IcsA::BIO]+[pIcsA_i248_]	This study
MYRM692	RMA2043 [pSU23-IcsA::BIO]+[pIcsA::BIO Y716G D717G]	This study
MYRM693	RMA2043 [pSU23-IcsA::BIO] +[pIcsA::BIO V382R]	This study
MYRM694	RMA2043 [pSU23-IcsA_i248_]+[pIcsA::BIO Y716G D717G]	This study
MYRM695	RMA2043 [pSU23-IcsA_i248_]+[pIcsA::BIO V382R]	This study
MYRM696	RMA2043 [pSU23-IcsA::BIO Y716G D717G]+[pIcsA_i248_]	This study
MYRM697	RMA2043 [pSU23-IcsA::BIO Y716G D717G]+[pIcsA::BIO V382R]	This study
MYRM699	RMA2043 [pBR322]+[pSU23-IcsA]	This study
MYRM700	RMA2043 [pBR322]+[pSU23-IcsA::BIO]	This study
MYRM701	RMA2043 [pBR322]+[pSU23-IcsA_i248_]	This study
MYRM702	RMA2043 [pBR322]+[pSU23-IcsA::BIO Y716G D717G]	This study
MYRM703	RMA2043 [pSU23]+[pIcsA]	This study
MYRM704	RMA2043 [pSU23]+[pIcsA::BIO]	This study
MYRM705	RMA2043 [pSU23]+[pIcsA_i248_]	This study
MYRM706	RMA2043 [pSU23]+[pIcsA::BIO Y716G D717G]	This study
MYRM707	RMA2043 [pSU23] +[pIcsA::BIO V382R]	This study
MYRM708	RMA2043 [pSU23-IcsA]+[pIcsA_i248_]	This study
MYRM709	RMA2043 [pSU23-IcsA]+[pIcsA::BIO Y716G D717G]	This study
MYRM710	RMA2043 [pSU23-IcsA] +[pIcsA::BIO V382R]	This study
**Plasmids**		
pBR322	medium copy no.; ColE1 *ori*; Ap^R^, Tc^R^	[44]
pSU23	medium copy no.; P15A *ori;* Cm^R^	[45]
pMG55	pBR322 encoding IcsA::BIO; Ap^R^	[19]
pIcsA	pBR322 encoding IcsA; Ap^R^	[43]
pIcsA_i248_	pIcsA with 5 amino acids insertion at aa 248; Ap^R^	[20]
pSU23-IcsA	pSU23 encoding IcsA; Cm^R^	This study
pSU23-IcsA::BIO	pSU23 encoding IcsA::BIO; Cm^R^	This study
pSU23-IcsA_i248_	pSU23 encoding IcsA_i248_; Cm^R^	This study
pSU23-IcsA::BIO Y716G D717G	pSU23 encoding IcsA::BIO Y716G D717G; Cm^R^	This study
pMYRM315	pMG55 Y716L D717S; Ap^R^	This study
pMYRM317	pMG55 Y716S D717K; Ap^R^	This study
pMYRM318	pMG55 Y716C D717C; Ap^R^	This study
pMYRM319	pMG55 Y716V D717H; Ap^R^	This study
pMYRM322	pMG55 Y716G D717G; Ap^R^	This study
pMYRM324	pMG55 Y716E D717I; Ap^R^	This study
pMYRM340	pMG55 Y716K D717S; Ap^R^	This study
pMYRM536	pMG55 T330S G331G; Ap^R^	This study
pMYRM537	pMG55 T330G G331R; Ap^R^	This study
pMYRM538	pMG55 T330E G331K; Ap^R^	This study
pMYRM539	pMG55 T330P G331G; Ap^R^	This study
pMYRM543	pMG55 T330G G331P; Ap^R^	This study
pMYRM556	pMG55 T330K G331N; Ap^R^	This study
pMYRM544	pMG55 T381Q V382R; Ap^R^	This study
pMYRM545	pMG55 T381R V382K; Ap^R^	This study
pMYRM546	pMG55 T381A V382Q; Ap^R^	This study
pMYRM547	pMG55 T381M V382L; Ap^R^	This study
pMYRM548	pMG55 T381K V382M; Ap^R^	This study
pMYRM549	pMG55 T381P V382R; Ap^R^	This study
pMYRM588	pMG55 G331W; Ap^R^	This study
pMYRM590	pMG55 V382R; Ap^R^	This study
pMYRM592	pMG55 Y716F; Ap^R^	This study
pMYRM595	pMG55 Y716G; Ap^R^	This study
pMYRM608	pMG55 D717G; Ap^R^	This study

a Tc^R^, Tetracycline resistant; Km^R^, Kanamycin resistant; Ap^R^, Ampicillin resistant; Cm^R^, Chloramphenicol resistant.

### Growth Media and Growth Conditions

All strains used in this study were routinely grown in Luria Bertani (LB) medium. *S. flexneri* strains were grown from a Congo Red positive colony as previously described [29]. Bacterial cultures were cultured for 18 h, diluted 1∶20 and grown to mid-exponential phase (2 h) with aeration at 37°C. Where appropriate, antibiotics were used at the following concentrations: ampicillin (Ap, 100 µg mL^−1^), chloramphenicol (Cm, 25 µg mL^−1^), kanamycin (Km, 50 µg mL^−1^), or tetracycline (Tet, 10 µg mL^−1^).

### DNA Methods

*E. coli* K-12 DH5α was used for all cloning. DNA manipulation, PCR, transformation and electroporation into *S. flexneri* were performed as previously described [30], [31].

### Antibodies and Antisera

Affinity purified rabbit polyclonal antibody to IcsA was produced as previously described [32]. The antiserum was produced under the National Health and Medical Research Council (NHMRC) Australian Code of Practice for the Care and Use of Animals for Scientific Purposes and was approved by the University of Adelaide Animal Ethics Committee. The anti-IcsA antibody was used at 1∶100 in immunofluorescence (IF) assay or 1∶1000 in Western immunoblotting (WB). The rabbit polyclonal anti-N-WASP [20] and monoclonal anti-Arp3 (BD Biosciences) antibodies were both used at 1∶100 in IF.

### Preparation of Whole Cell Lysate

The equivalent of 5×10^8^ bacteria were centrifuged (2,200 x*g*, 1 min) and resuspended in 100 µl of 1x sample buffer [33]. Samples were heated to 100°C for 5 min prior to electrophoresis.

### Western Transfer and Detection

Western immunoblotting was performed as described previously [20] with some modifications. Briefly, proteins were separated on sodium dodecyl sulfate-7.5% or 12%-polyacrylamide gel electrophoresis (SDS-PAGE) gels and transferred to a nitrocellulose membrane. The membrane was blocked with 5% (w/v) skim milk in TTBS (Tris-buffered saline, 0.005% (v/v) Tween-20) for 20 min and incubated with anti-IcsA antibody in TTBS containing 2.5% (w/v) skim milk for 18 h. After three washes in TTBS, the membrane was incubated with horseradish peroxidise-conjugated goat anti-rabbit secondary antibody (Biomediq DPC) for 2 h, followed by three washes in TTBS, then three times in TBS. The membrane was incubated with CPS3 chemiluminescence substrate (Sigma) for 5 min, followed by exposure of the membrane to either X-ray film (Agfa) or imaged with a Kodak Image Station 4000 MM Pro (Carestream Molecular Imaging), to visualise the reactive bands. The film was developed using a Curix 60 automatic X-ray film processor (Agfa).

### Site-directed Mutagenesis

Single and multi site-directed mutagenesis were performed according to the QuikChange® Lightning Site-directed or QuikChange® Lightning Multi Site-directed protocol (Stratagene), respectively. Specific primers with the desired substitutions were designed for single site-directed mutagenesis (Table S1) and degenerate primers were designed for multi site-directed mutagenesis (Table S1). pMG55 (pIcsA::BIO) was used as the DNA template for the construction of the mutants. IcsA::BIO protein possesses a BIO epitope (GLNDIFEAQKIEWH; a substrate for metabolic biotinylation by the BirA biotin-protein ligase [34]) introduced at aa 87 which does not affect the IcsA production levels, polar localisation and function [19]. Briefly, the resultant PCR products were treated with *Dpn*I and transformed into XL 10-Gold ultracompetent cells (Stratagene). Mutations were confirmed by DNA sequencing.

### Construction of pSU23-IcsA Plasmids

pSU23-IcsA_WT_, pSU23-IcsA::BIO, pSU23-IcsA_i248_ and pSU23-IcsA::BIO Y716G D717G were constructed by sub-cloning the *Pst*I-*Sal*I fragment of pIcsA_WT_, pIcsA::BIO, pIcsA_i248_ and pIcsA::BIO Y716G D717G (pMYRM322), encoding different IcsA proteins, respectively, into likewise digested pSU23.

### Plaque Assays

Plaque assays were performed as described previously [20] using the method of Oaks *et al*. [35]. Briefly, HeLa cells (Human, cervical, epithelial cells ATCC #CCL-70) were grown to confluence overnight in minimal essential medium (MEM)-10% (v/v) foetal calf serum (FCS), washed twice with Dulbecco’s PBS (D-PBS) and once in Dulbecco’s modified Eagle medium (DMEM) prior to infection. 1×10^8^ bacteria mL^−1^ mid-exponential phase bacteria were diluted to 1∶1000 in DMEM, and 0.25 mL was added to each well. Trays were incubated in a CO_2_ (5%) incubator, and the trays were gently rocked every 15 min. At 120 min post-infection, the inoculum was carefully aspirated and 3 mL of the first overlay (DMEM, 5% (v/v) FCS, 20 µg mL^−1^ gentamycin, 0.5% (w/v) agarose [Seakem ME]) was added to each well. The second overlay (DMEM, 5% (v/v) FCS, 20 µg mL^−1^ gentamycin, 0.5% (w/v) agarose, 0.1% (w/v) Neutral Red solution [Gibco BRL]) was added at either 24 or 48 h post-infection and plaque formation observed 6–8 h later.

### Indirect Immunofluorescence of Whole Bacteria

Indirect IF labelling of bacteria was performed as previously described [20]. Briefly, mid-exponential phase bacteria were fixed in formalin (3.7% (v/v) paraformaldehyde in 0.85% (w/v) saline) and centrifuged onto poly-L-lysine-coated coverslips. Bacteria were incubated with anti-IcsA antibody, washed with PBS and labelled with Alexa 488-conjugated donkey anti-rabbit secondary antibody (Molecular Probes) (1∶100). Microscopy was performed as previously described [20], using an Olympus IX-70 microscope with phase-contrast optics using a 100x oil immersion objective. Fluorescence and phase-contrast images were false colour merged using the Metamorph software program (Version 7.7.3.0, Molecular Devices).

### Infection of Tissue Culture Monolayers with S. Flexneri and IF Labelling

Infection of HeLa cell monolayers and IF staining were performed as previously described [20]. HeLa monolayers were inoculated with mid-exponential phase bacteria and incubated for 1 h at 37°C, CO_2_ (5%). The infected monolayers were washed three times with D-PBS and incubated with MEM containing gentamycin for a further 1.5 h. Infected cells were then washed and fixed for 15 min in 3.7% (v/v) formalin, incubated with 50 mM NH_4_Cl in D-PBS for 10 min, and permeabilised with 0.1% (v/v) Triton X-100 for 5 min. The infected cells were blocked with 10% (v/v) FCS in PBS and incubated with the desired primary antibody at 37°C for 30 min. After washing in PBS, coverslips were incubated with Alexa Fluor 594-conjugated donkey anti-rabbit, Alexa Fluor 488-conjugated donkey anti-rabbit or Alexa Fluor 594-conjugated donkey anti-mouse secondary antibodies (Molecular Probes) (1∶100), where appropriate. F-actin was visualised by staining with Alexa Fluor 488-conjugated phalloidin (2 U mL^−1^), and 4′,6′-diamidino-2-phenylindole (DAPI) (10 µg mL^−1^) was used to counterstain bacteria and cellular nuclei.

## Results

### N-WASP Interacting Region II

May & Morona [20] previously identified a region in IcsA (aa 330–382) that was involved in N-WASP recruitment, based on linker-insertion mutagenesis. Protein production of IcsA_i_ mutants with insertion mutations within this region was unaffected but the mutants were defective in ABM when expressed in *S. flexneri* Δ*icsA* S-LPS [20]. This region was classified as N-WASP IR II. Since the 5 aa linker insertion may disrupt local protein structure conformation (IcsA_i330_ and IcsA_i381_), it was of interest to determine if altering specific residues would reproduce the same phenotype, thus, enabling the identification of specific amino acids involved in N-WASP recruitment within this region. Multi site-directed mutagenesis was performed on pIcsA::BIO (pMG55) to randomly substitute both amino acids, 330–331 and 381–382, respectively, by using degenerate primers listed in Table S1. Mutants were isolated, DNA sequenced, and listed in Tables 2 and 3. IcsA::BIO T330*G331* and IcsA::BIO T381*V382* mutant production in *S. flexneri* Δ*icsA* was assessed by Western immunoblotting with anti-IcsA antibody (data not shown). All of these IcsA mutant proteins were produced by *S. flexneri* at WT levels (Tables 2 and 3).

**Table 2:** Summary of *S. flexneri* strains expressing IcsA::BIO T330*G331* and IcsA::BIO G331W.

Plasmid	Codon#330–331	Amino acids	Polarity^a^	IcsA production^b^	N-WASP recruitment*^c^*	F-actin tails*^d^*	Plaque formation^e^
pIcsA::BIO	ACT GGT	Thr Gly	P, P	+++	+++	+++	+++
pMYRM536	AGC GGG	Ser Gly	P, P	+++	+++	+++	+++
pMYRM537	GGG AGG	Gly Arg	P, Basic	+++	+/−	+/−	−
pMYRM538	GAG AAG	Glu Lys	Acidic, Basic	+++	++	++	++
pMYRM539	CCG GGC	Pro Gly	NP, P	+++	−	−	−
pMYRM543	GGG CCG	Gly Pro	P, NP	+++	−	−	−
pMYRM556	AAG AAC	Lys Asn	Basic, P	+++	++	++	++
pMYRM588 (pIcsA::BIO G331W)	ACT TGG	Thr Trp	P, NP	+++	++	++	++

a P, polar; NP, non-polar.

b The “+++” symbol indicates relative band intensities of Western immunoblots of whole cell lysates.

c, d “+++”, WT N-WASP recruitment/F-actin comet tail or capping formation; “++”, 20%–80% reduction in N-WASP recruitment/F-actin comet tail or capping formation; “+/−”, 90% reduction in N-WASP recruitment/F-actin comet tail or capping formation; “−”, N-WASP/F-actin tail not detected.

e “+++”, WT plaques; “++”, small plaques; “−”, no plaques.

**Table 3:** Summary of *S. flexneri* strains expressing IcsA::BIO T381*V382* and IcsA::BIO V382R.

Plasmid	Codon#381–382	Amino acids	Polarity^a^	IcsAproduction^b^	N-WASP recruitment*^c^*	F-actin tails*^d^*	Plaque formation^e^
pIcsA::BIO	ACT GTT	Thr Val	P, NP	+++	+++	+++	+++
pMYRM544	CAG CGG	Gln Arg	P, Basic	+++	−	−	−
pMYRM545	CGG AAG	Arg Lys	Basic, Basic	+++	−	−	−
pMYRM546	GCG CAG	Ala Gln	NP, P	+++	−	−	−
pMYRM547	ATG TTG	Met Leu	NP, NP	+++	+++	+++	+++
pMYRM548	AAG ATG	Lys Met	Basic, NP	+++	++	++	++
pMYRM549	CCC CGC	Pro Arg	NP, Basic	+++	−	−	−
pMYRM590 (pIcsA::BIO V382R)	ACT CGT	Thr Arg	P, Basic	+++	−	−	−

a P, polar; NP, non-polar.

b The “+++” symbol indicates relative band intensities of Western immunoblots of whole cell lysates.

c, d “+++”, WT N-WASP recruitment/F-actin comet tail formation; “++”, 20%–80% reduction in N-WASP recruitment/F-actin comet tail or capping formation; “−”, N-WASP/F-actin comet tail not detected.

e “+++”, WT plaques; “++”, small plaques; “−”, no plaques.

The ability of *S. flexneri* Δ*icsA* expressing IcsA::BIO T330*G331* or IcsA::BIO T381*V382* mutants to recruit N-WASP and form F-actin comet tail was investigated by IF microscopy using anti-N-WASP antibody and Alexa Fluor 488-phalloidin, respectively (data not shown). The results are summarised in Tables 2 and 3. T330 and G331 are both polar amino acids, and IcsA::BIO T330*G331* mutants that have at least one non-polar amino acid (proline) substitution at either position (e.g. encoded by pMYRM539 and pMYRM543) did not recruit N-WASP (Table 2). Notably, mutants encoded by pMYRM536 [IcsA::BIO **T330S** G331G] and pMYRM539 [IcsA::BIO **T330P** G331G] both possess single mutations at residue 330 **(in bold)** but only mutant T330S G331G (both polar amino acids) was capable of recruiting N-WASP. However, it cannot be ruled out that the proline substitution at position 330 or 331 is causing a localised conformation change thereby affecting IcsA function. Furthermore, mutants with charged amino acids (encoded by pMYRM537, pMYRM538 and pMYRM556) recruited less N-WASP and formed less F-actin capping/tails (Table 2), suggesting that polar residues are required for efficient N-WASP recruitment.

In contrast, T381 and V382 are polar and non-polar amino acids, respectively. Based on the IF microscopy results (Table 3), IcsA::BIO T381*V382* mutants with residue 382 substituted with either charged or polar amino acids (e.g. encoded by pMYRM544, pMYRM545, pMYRM546 and pMYRM549) were unable to recruit N-WASP, whilst mutants that had V382 substituted with another non-polar amino acid (e.g. encoded by pMYRM547 and pMYRM548) recruited N-WASP, regardless of the amino acid polarity at residue 381. The data suggest that a non-polar amino acid is required at residue 382 in order for IcsA to be capable of recruiting N-WASP.

IcsA::BIO T330*G331* mutants that recruited N-WASP were selected to examine their ability to perform intercellular spreading by plaque assay (Fig. S1). As expected, T330*G331* that recruited N-WASP formed plaques but with different plaque sizes, depending on the amino acids substitution. Similar results were obtained for IcsA::BIO T381*V382* mutants wherein mutants that had non-polar amino acids substituted at residue 382, and also recruited N-WASP, formed plaques. In all cases, there was a consistent correlation between N-WASP recruitment, F-actin comet tail/capping formation and plaque formation. Taken together, the data suggest that both residues 330–331 of IcsA need to be polar while residue 382 needs to be non-polar in order for IcsA to be able to recruit N-WASP.

### Effect of the G331W Mutation on N-WASP Recruitment and Intercellular Spreading

**Figure 1:**
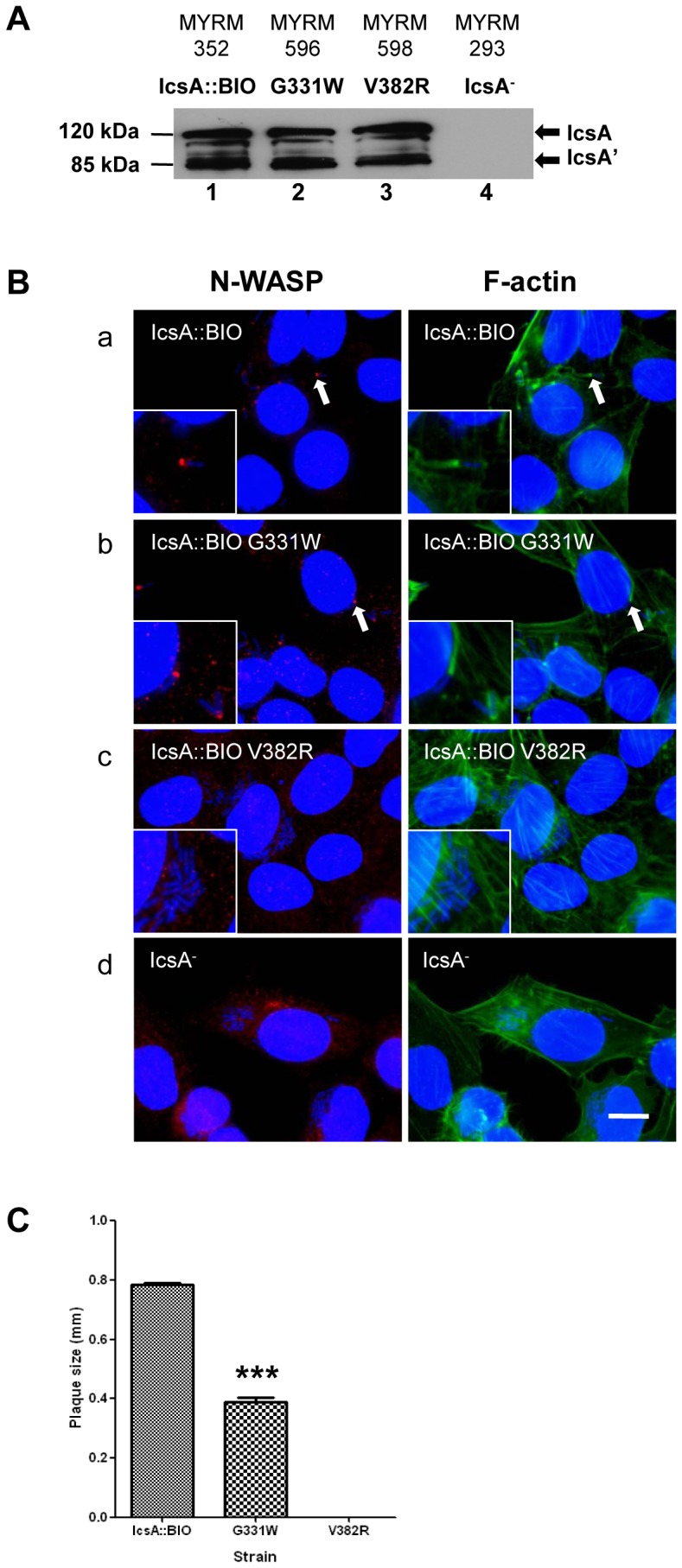
Expression of IcsA::BIO G331W and IcsA::BIO V382R and effect on N-WASP recruitment and intercellular spreading. (A) Whole cell lysates from mid-exponential phase cultures of the indicated *S. flexneri* strains were subjected to Western immunoblotting with anti-IcsA antibody. S = S-LPS; R = R-LPS. IcsA^−^ = IcsA deletion control. The 120 kDa band corresponds to the full length IcsA and the 85 kDa band corresponds to the cleaved form (IcsA’). (B) HeLa cells were infected with mid-exponential phase *S. flexneri ΔicsA* expressing the IcsA mutants and formalin fixed. HeLa cells and bacteria nuclei were labelled with DAPI (blue), F-actin was labelled with Alexa Fluor 488-phalloidin (green), and N-WASP was labelled with anti-N-WASP antibody and Alexa Fluor 594-conjugated donkey anti-rabbit antibody (red) as detailed in Materials and Methods. IF images were observed at 100×magnification. Arrows indicate N-WASP recruitment and F-actin comet tail formation. Insert shows an enlargement of the indicated region. Strains were assessed in two independent experiments. Scale bar = 10 µm. (C) Plaque assay by *S. flexneri ΔicsA* strains expressing IcsA::BIO, IcsA::BIO G331W or IcsA::BIO V382R. Confluent HeLa cell monolayers were infected with mid-exponential phase *S. flexneri* strains for 2 h, and plaques were observed 48 h post-infection as detailed in Materials and Methods. 30 plaques were measured from each experiment. Data are represented as mean ± SEM of three independent experiments. ***, *P*<0.001 (determined by Student’s unpaired one-tailed *t* test).

We hypothesised that residues 330–331 require the polar-polar combination to recruit N-WASP. Thus, site-directed mutagenesis was performed on pIcsA::BIO using primer pairs listed in Table S1 to mutate G331 to tryptophan (W), which is a non-polar, aromatic amino acid. IcsA::BIO G331W (encoded by pMYRM588) was expressed in *S. flexneri* Δ*icsA*, and protein production was examined by Western immunoblotting (Fig. 1A, lane 2) and IF microscopy (Fig. S2). IcsA::BIO G331W was expressed on the bacterial surface and was produced at a WT comparable levels. As seen by IF microscopy, IcsA::BIO G331W mutant was capable of recruiting N-WASP and formed F-actin comet tails but seemed less frequent than the functionally equivalent IcsA::BIO (Fig. 1B(b)). Its ability to promote intercellular spreading was also tested by plaque assay. Plaques formed by IcsA::BIO G331W were half the size (***P<0.001) of those formed by *S. flexneri* expressing IcsA::BIO (Fig. 1C). Collectively, the data suggest that the G331W mutation affected intercellular spreading of *S. flexneri*, presumably by affecting N-WASP recruitment efficiency. In addition, the data also indicate that a polar-polar combination for residues 330–331 is not essential for N-WASP recruitment.

### Effect of the V382R Mutation on N-WASP Recruitment and Intercellular Spreading

We hypothesised that it is essential for IcsA residue 382 to be a non-polar amino acid for N-WASP recruitment. Site-directed mutagenesis was performed on pIcsA::BIO to mutate V382 to arginine (R) that is positively charged. IcsA::BIO V382R (encoded by pMYRM590) mutant production by *S. flexneri* Δ*icsA* was assessed by Western immunoblotting (Fig. 1A, lane 3) and IF microscopy (Fig. S2). IcsA::BIO V382R was expressed on the bacterial surface and produced at a WT comparable levels. Its ability to recruit N-WASP and form F-actin comet tails within HeLa cells was examined. IF microscopy showed that the V382R mutant was defective in N-WASP recruitment and F-actin comet tail formation (Fig. 1B(c)). Consequently, *S. flexneri* expressing the IcsA::BIO V382R mutant was unable to form plaques (Fig. 1C). These data suggest that a non-polar amino acid (aa 382) is a prerequisite for N-WASP recruitment and residue 382 is likely to be an N-WASP binding/interaction site.

### N-WASP Interacting Region III

**Figure 2:**
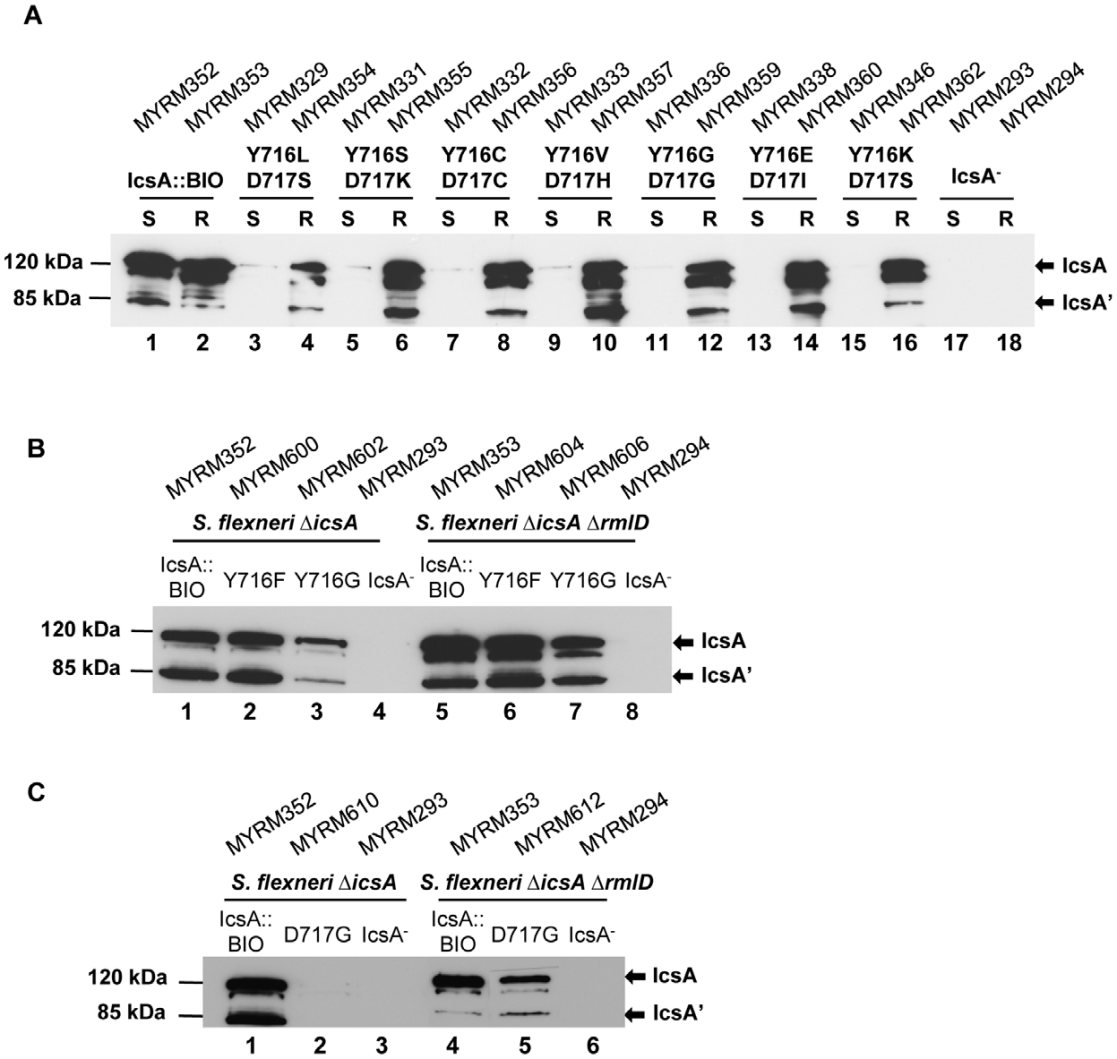
Expression of IcsA::BIO with mutations at Y716 D717 by S-LPS and R-LPS *S. flexneri*. (A) Whole cell lysates from mid-exponential phase *S. flexneri ΔicsA* (S-LPS) and *S. flexneri* Δ*icsA* Δ*rmlD* (R-LPS) expressing (A) IcsA::BIO or various IcsA::BIO Y716* D717* mutants; (B) IcsA::BIO, IcsA::BIO Y716F or IcsA::BIO Y716G; (C) IcsA::BIO or IcsA::BIO D717G; were prepared and analysed by Western immunoblotting using anti-IcsA antibody. *S. flexneri* carrying an empty vector was used as a negative control (IcsA^−^). Strain names are shown above each lane. The 120 kDa band corresponds to the full length IcsA; the 85 kDa band corresponds to the cleaved form (IcsA’). S = S-LPS; R = R-LPS.

We have previously characterised several IcsA_i_ mutants which had 5 aa insertion mutations within the IcsA AC region for their ability to recruit N-WASP, and identified that IcsA_i716_ was defective in N-WASP recruitment [28]. These IcsA_i_ mutants had extremely low protein production in the S-LPS background but WT comparable expression levels in the R-LPS background [28]. The IcsA_i716_ mutant has a 5 aa insertion mutation after residue 716 which affects the Aro-X-Aro (aromatic residue-any-aromatic residue) motif, Y_716_-D_717_-Y_718_. This motif is commonly found in the β-domain of ATs and is thought to be a preferential binding site of SurA [36], [37]. Hence, it was of interest to determine if altering residues 716 and 717 would reproduce the effect of the insertion mutation that may have an effect on local protein conformation, in terms of IcsA biogenesis and N-WASP recruitment ability. The recently identified IcsA AC crystal structure (Fig. S3) shows that the predicted locations for residues 716–717 appeared to be surface exposed, while residue 718 is predicted to be buried. Hence, multi site-directed mutagenesis was performed on pIcsA::BIO to substitute both Y716 and D717 with random amino acids at these positions. Seven pIcsA::BIO Y716*D717* mutants were isolated, DNA sequenced, and are listed in Table 4. The production of IcsA::BIO Y716*D717* mutants in both *S. flexneri* Δ*icsA* S-LPS and *S. flexneri* Δ*icsA* Δ*rmlD* R-LPS strains was determined by Western immunoblotting. The IcsA::BIO Y716*D717* mutant production was markedly reduced in the S-LPS strain, regardless of the amino acids substitutions (Fig. 2A). As expected, similar to the IcsA_i_ mutants [28], IcsA::BIO Y716*D717* mutant production in the R-LPS strain was restored to a level that was comparable to IcsA::BIO (Fig. 2A).

**Table 4:** Analysis of *S. flexneri* strains expressing IcsA::BIO Y716* and/or D717* in S-LPS and R-LPS backgrounds.

Plasmid	Codon#716–717	Aminoacids	Polarity^a^	IcsA production^b^		N-WASP recruitment*^c^*		F-actin tails*^d^*		Plaque Formation^e^
				S-LPS	R-LPS	S-LPS	R-LPS	S-LPS	R-LPS	
pIcsA::BIO	TAT GAC	Tyr Asp	NP, Acidic	+++	+++	+++	+++	+++	+++	+++
pMYRM315	TTG AGC	Leu Ser	NP, P	+	+++	−	−	−	−	−
pMYRM317	AGC AAG	Ser Lys	P, Basic	+	+++	−	−	−	−	−
pMYRM318	TGC TGC	Cys Cys	NP, NP	+	+++	−	−	−	−	−
pMYRM319	GTG CAC	Val His	NP, Basic	+	+++	−	−	−	−	−
pMYRM322	GGG GGC	Gly Gly	P, P	+	+++	−	−	−	−	−
pMYRM324	GAG ATC	Glu Ile	Acidic, NP	+	+++	−	−	−	−	−
pMYRM340	AAG ATC	Lys Ser	Basic, P	+	+++	−	−	−	−	−
pMYRM592 (pIcsA::BIO Y716F)	TTT GAC	Phe Asp	NP, Acidic	+++	+++	+++	+++	+++	+++	+++
pMYRM595 (pIcsA::BIO Y716G)	GGT GAC	Gly Asp	P, Acidic	++	++	++	+++	++	+++	++
pMYRM608 (pIcsA::BIO D717G)	TAT GGC	Tyr Gly	NP, P	+	++	−	+/−	−	+/−	−

a P, polar; NP, non-polar.

b The “+++”, “++” and “+” symbols indicate relative band intensities of Western immunoblots of whole cell lysates.

c, d “+++”, WT N-WASP recruitment/F-actin comet tail or capping formation; “++”, 20%–80% reduction in N-WASP recruitment/F-actin comet tail or capping formation; “+/−”, 90% reduction in N-WASP recruitment/F-actin comet tail or capping formation; “−”, N-WASP/F-actin tail not detected.

e “+++”, WT plaques; “++”, small plaques; “−”, no plaques.

We then investigated the effect of individual residues (Y716 and D717) by performing site-directed mutagenesis on pIcsA::BIO to mutate Y716 to phenylalanine (F) or glycine (G), and D717 to glycine (G) using primer pairs shown in Table S1. Plasmid constructs pIcsA::BIO Y716F (pMYRM592), pIcsA::BIO Y716G (pMYRM595) and pIcsA::BIO D717G (pMYRM608) were transformed into both *S. flexneri* Δ*icsA* S-LPS and *S. flexneri* Δ*icsA* Δ*rmlD* R-LPS strains and protein production was examined by Western immunoblotting (Fig. 2B) and IF microscopy (Fig. S4). The Y716F mutation did not affect IcsA production (Fig. 2B, lanes 2 and 6) but the Y716G mutation (Fig. 2B, lanes 3 and 7) resulted in reduced IcsA production, in both S-LPS and R-LPS backgrounds. Similar to other IcsA mutants with mutations within the AC region [28], the D717G mutation greatly reduced IcsA production in *S. flexneri* Δ*icsA* S-LPS (Fig. 2C, lane 2) but the production was restored in *S. flexneri* Δ*icsA* Δ*rmlD* R-LPS, albeit slightly lower than IcsA::BIO (Fig. 2C, lane 5). Hence, whereas aa 717 is essential for IcsA biogenesis, the aromatic aa 716 is also important but less dependent on LPS structure.

### Effect of Y716 and D717 Mutagenesis on N-WASP Recruitment and F-actin Comet Tail Formation

We have previously shown that IcsA_i716_ was defective in N-WASP recruitment and proposed that residues 716–717 could be an N-WASP binding/interaction site [28]. To clarify this hypothesis, the IcsA::BIO Y716*D717* mutants, IcsA::BIO Y716F, IcsA::BIO Y716G and IcsA::BIO D717G mutants were assessed for their ability to recruit N-WASP and form F-actin comet tail by IF microscopy (in both S-LPS and R-LPS backgrounds). The results are summarised in Table 4. For the IcsA::BIO Y716*D717* mutants, regardless of the amino acids substitutions, none of the mutants recruited N-WASP in HeLa cells (in both S-LPS and R-LPS backgrounds), despite the higher protein expression levels in the R-LPS background (Fig. 2A), which suggest that residues 716–717 are an N-WASP binding/interaction site.

**Figure 3:**
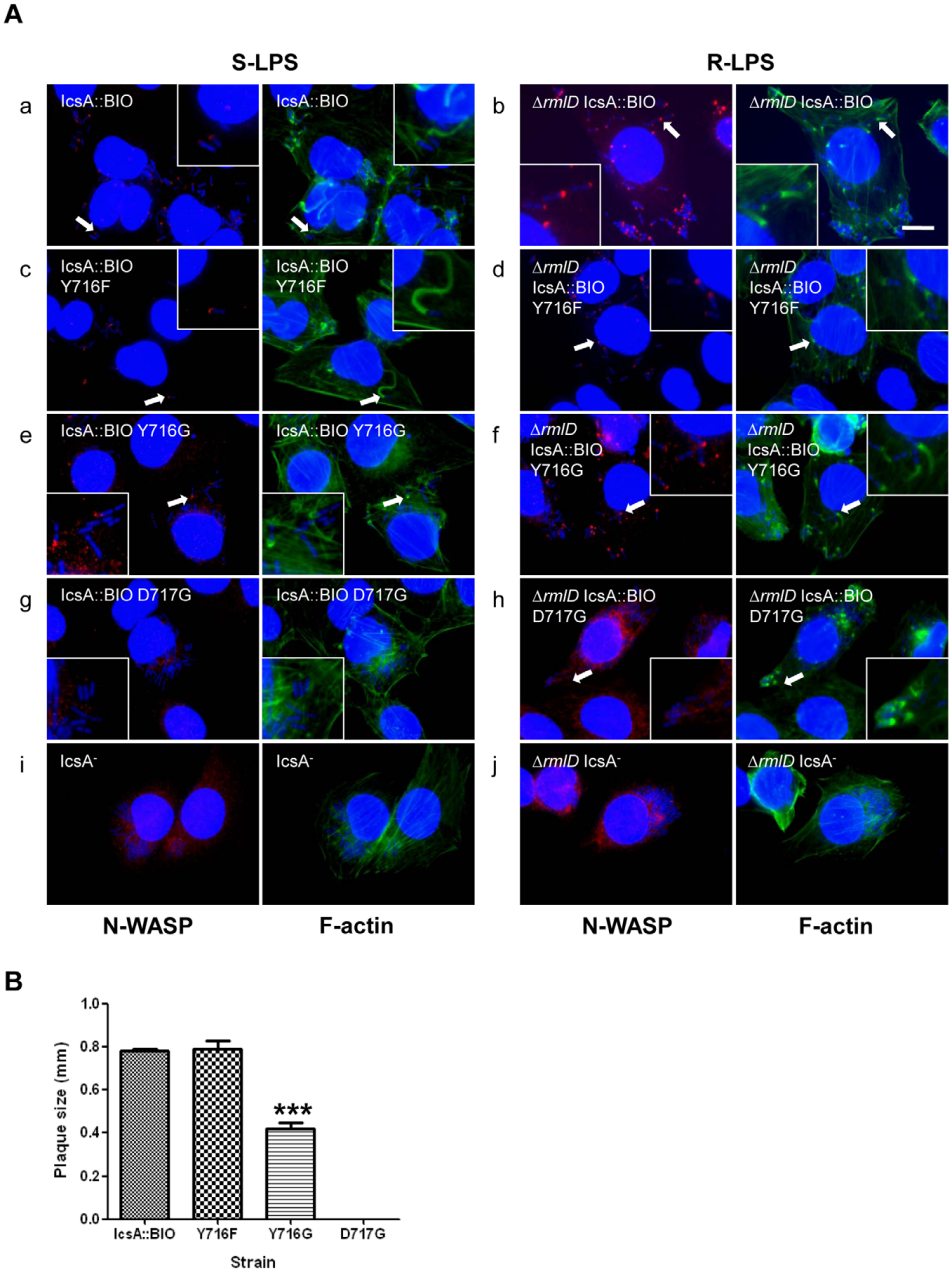
Effect of mutagenesis of IcsA::BIO Y716 and IcsA::BIO D717 on N-WASP recruitment and intercellular spreading. (A) HeLa cells were infected with mid-exponential phase *S. flexneri ΔicsA* (S-LPS) or *S. flexneri* Δ*icsA* Δ*rmlD* (R-LPS) having an empty vector or expressing IcsA::BIO, IcsA::BIO Y716F, IcsA::BIO Y716G or IcsA::BIO D717G, and then formalin fixed. HeLa cells and bacteria nuclei were labelled with DAPI (blue), F-actin was labelled with Alexa Fluor 488-phalloidin (green), and N-WASP was labelled with anti-N-WASP antibody and Alexa Fluor 594-conjugated donkey anti-rabbit antibody (red) as detailed in Materials and Methods. IF images were observed at 100×magnification. Arrows indicate N-WASP recruitment and F-actin comet tail formation. Enlargements of relevant region shown for clarity. Strains were assessed in two independent experiments. Scale bar = 10 µm. (B) Plaque assay of *S. flexneri ΔicsA* strains expressing IcsA::BIO, IcsA::BIO Y716F, IcsA::BIO Y716G or IcsA::BIO D717G. Confluent HeLa cell monolayers were infected with mid-exponential phase *S. flexneri* strains for 2 h, and plaques were observed 48 h post-infection as detailed in Materials and Methods. 30 plaques were measured from each experiment. Data are represented as mean ± SEM of three independent experiments. ***, *P*<0.001 (determined by Student’s unpaired one-tailed *t* test).

Investigation of individual residues, Y716 and D717, on N-WASP recruitment showed that *S. flexneri* Δ*icsA* S-LPS strain expressing IcsA::BIO Y716F recruited N-WASP and formed F-actin comet tails (Fig. 3A(c)), while the *S. flexneri* Δ*icsA* S-LPS strain expressing IcsA::BIO Y716G recruited N-WASP (slightly less than IcsA::BIO) but had only F-actin capping formation and F-actin comet tails were not observed (Fig. 3A(e)). However, both Y716F and Y716G mutants expressed by the *S. flexneri* Δ*icsA* Δ*rmlD* R-LPS strains recruited N-WASP and formed F-actin comet tails (Fig. 3A(d,f)), suggesting that the aromatic property of residue 716 is not essential for N-WASP binding/interaction. As expected, *S. flexneri* Δ*icsA* S-LPS expressing IcsA::BIO D717G did not recruit any N-WASP or form F-actin comet tail, likely due to the very low protein level (Fig. 3A(g)). In contrast, despite the higher level of protein production in the R-LPS background, N-WASP staining was barely detectable and F-actin capping was detected on a minor population of *S. flexneri* Δ*icsA* Δ*rmlD* expressing IcsA::BIO D717G (∼15%) (Fig. 3A(h)). Collectively, the data suggest that residue D717 has a greater impact on N-WASP recruitment in comparison to Y716.

### Effect of the Y716F, Y716G or D717G Mutation on IcsA Function in Intercellular Spreading

The ability of *S. flexneri* Δ*icsA* S-LPS expressing IcsA::BIO Y716F, IcsA::BIO Y716G and IcsA::BIO D717G to perform ABM was investigated by assaying plaque formation. While *S. flexneri* Δ*icsA* [IcsA::BIO Y716F] formed plaques comparable to the WT control (IcsA::BIO), *S. flexneri* Δ*icsA* [IcsA::BIO Y716G] formed plaques that were 50% smaller (***P<0.001) (Fig. 3B). These results are consistent with the IF microscopy data whereby *S. flexneri* Δ*icsA* expressing IcsA::BIO Y716G only formed F-actin capping, and hence were affected in ABM efficiency. As expected, *S. flexneri* Δ*icsA* [IcsA::BIO D717G] that does not recruit N-WASP due to very low protein expression, did not form plaques (Fig. 3B).

### Co-expression of Mutated IcsA Proteins in S. flexneri

Our results and those reported by May & Morona [20] showed that N-WASP recruitment was partly affected or completely abolished when IcsA protein with mutation within any of the N-WASP IRs (I, II or III) was expressed by *S. flexneri* (summary in Table S2). We have also recently found that IcsA is able to self-associate [19]. Therefore, we hypothesised that all three N-WASP interacting regions are required for N-WASP binding/activation and co-expression of two IcsA proteins with mutations at different N-WASP interacting regions would be able to complement the N-WASP binding ability and ultimately, restore N-WASP recruitment and activation. To co-express two mutated IcsA proteins, relevant *icsA* genes were cloned into vector pSU23 which has a similar copy number (intermediate) to pBR322. IcsA mutants with mutation at each N-WASP IR and defective in N-WASP recruitment when expressed individually in *S. flexneri* were chosen for co-expression/complementation analysis. pSU23 constructs that encode for IcsA_WT_, IcsA::BIO, IcsA_i248_ (N-WASP IR I) and IcsA::BIO Y716G D717G (N-WASP IR III) were constructed as detailed in Materials and Methods. As expected, all IcsA proteins were expressed at WT levels that were comparable to those expressed by the pBR322 constructs in both S-LPS and R-LPS backgrounds (Fig. S5A), except IcsA::BIO Y716G D717G (N-WASP IR III) that had extremely low protein production in the S-LPS background (Fig. S5A, lane 7), which was similar to that observed for the pBR322 based clone (Fig. 2A). Similar results were obtained for IF microscopy where surface IcsA expression was detected at WT levels, except for IcsA::BIO Y716G D717G in the S-LPS background, as expected (Fig. S5B).

To co-express IcsA proteins, various pBR322-IcsA constructs that encode for IcsA_WT_, IcsA::BIO, IcsA_i248_ (N-WASP IR I), IcsA::BIO V382R (N-WASP IR II) or IcsA::BIO Y716G D717G (N-WASP IR III) were subsequently electroporated into different *S. flexneri* strains (S-LPS and R-LPS) carrying the pSU23-IcsA constructs as shown in Tables 5 and 6. *S. flexneri* S-LPS strains that co-expressed IcsA proteins were assayed for N-WASP recruitment following infection of HeLa cells by IF microscopy, and the results are summarised in Table 5. *S. flexneri* S-LPS strains that expressed either IcsA_WT_ or the functionally equivalent IcsA::BIO were able to recruit N-WASP, regardless of the co-expressed IcsA protein. However, co-expression of two mutated IcsA proteins (N-WASP IR I+II, II+III or I+III) did not result in N-WASP recruitment. As expected, *S. flexneri* strains that co-expressed a mutated IcsA protein with an empty vector did not recruit N-WASP. As the production of IcsA::BIO Y716G D717G was extremely low in the S-LPS background but restored to a WT comparable levels in the R-LPS background, various IcsA proteins were also co-expressed in *S. flexneri* R-LPS. Their ability to recruit N-WASP was examined and summarised in Table 6. The same outcomes were obtained for the *S. flexneri* R-LPS strains which have the same IcsA co-expression combination in the S-LPS background, with the remarkable exception of the co-expressed combination IcsA::BIO V382R and IcsA::BIO Y716G D717G (N-WASP IR II and III, respectively) that was able to recruit N-WASP (Table 6).

**Table 5:** N-WASP recruitment by *S. flexneri* S-LPS strains co-expressing various IcsA proteins.

				N-WASP IR I	N-WASP IR II	N-WASP IR III	
	Protein	IcsA_WT_ a	IcsA::BIO^a^	IcsA_i248_ a	IcsA::BIO V382R^a^	IcsA::BIO Y716G D717G^a^	pBR322 vector^a^
	**IcsA_WT_** b	nt	nt	✓	✓	✓	✓
	**IcsA::BIO** b	nt	nt	✓	✓	✓	✓
***N-WASP IR I***	**IcsA_i248_** b	nt	nt	nt	✗	✗	✗
***N-WASP IR III***	**IcsA::BIO Y716G D717G** b	nt	nt	✗	✗	nt	✗
	**pSU23 vector** b	✓	✓	✗	✗	✗	nt

a IcsA proteins expressed by pBR322 intermediate copy plasmid.

b IcsA proteins expressed by pSU23 intermediate copy plasmid.

“✓” = N-WASP recruited.

“✗” = No N-WASP recruited.

nt = not tested.

**Table 6:** N-WASP recruitment by *S. flexneri* R-LPS strains co-expressing various IcsA proteins.

				N-WASP IR I	N-WASP IR II	N-WASP IR III	
	Protein	IcsA_WT_ a	IcsA::BIO^a^	IcsA_i248_ a	IcsA::BIO V382R^a^	IcsA::BIO Y716G D717G^a^	pBR322 vector^a^
	**IcsA_WT_** b	nt	nt	✓	✓	✓	✓
	**IcsA::BIO** b	nt	nt	✓	✓	✓	✓
***N-WASP IR I***	**IcsA_i248_** b	nt	nt	nt	✗	✗	✗
***N-WASP IR III***	**IcsA::BIO Y716G D717G** b	nt	nt	✗	✓	nt	✗
	**pSU23 vector** b	✓	✓	✗	✗	✗	nt

a IcsA proteins expressed by pBR322 intermediate copy plasmid.

b IcsA proteins expressed by pSU23 intermediate copy plasmid.

“✓” = N-WASP recruited.

“✗” = No N-WASP recruited.

nt = not tested.

### N-WASP Activation by the Co-expressed IcsA::BIO V382R and IcsA::BIO Y716G D717G

**Figure 4:**
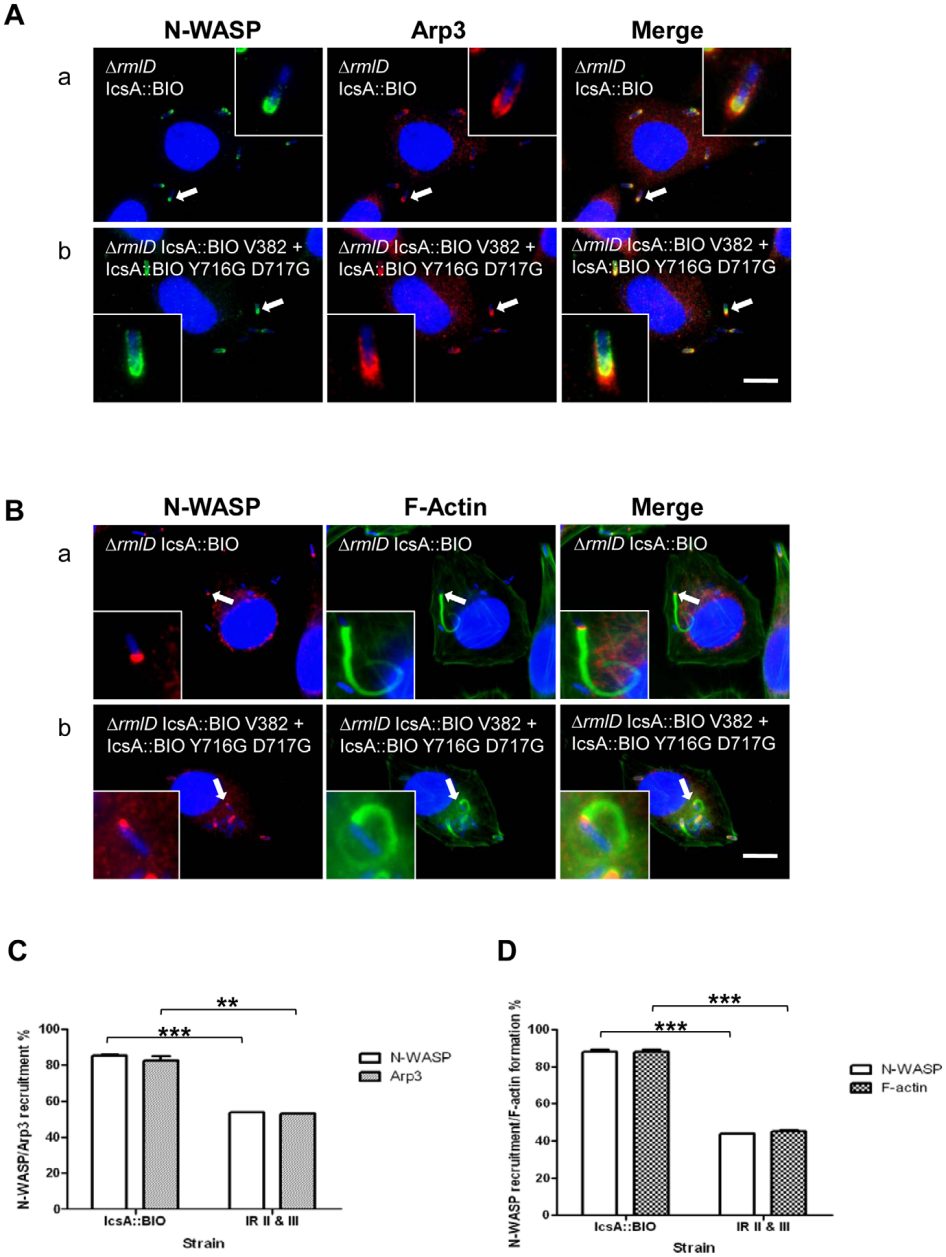
N-WASP interacting region complementation assay. (A, B) IF microscopy to detect N-WASP, Arp3 recruitment and F-actin comet tail formation by intracellular *S. flexneri* strains. HeLa cells were infected with mid-exponential phase *S. flexneri ΔicsA ΔrmlD* (R-LPS) strains expressing either IcsA::BIO only or co-expressing IcsA::BIO V382R (N-WASP IR II) and IcsA::BIO Y716G D717G (N-WASP IR III), and formalin fixed. HeLa cells and bacteria nuclei were labelled with DAPI (blue), and N-WASP was labelled with anti-N-WASP antibody and either (A) Alex Fluor 488-conjugated donkey anti-rabbit antibody (green) or (B) Alex Fluor 594-conjugated donkey anti-rabbit antibody (red). (A) Arp3 was labelled with anti-Arp3 monoclonal antibody and an Alex Fluor 594-conjugated donkey anti-mouse antibody (red). (B) F-actin was labelled with Alexa Fluor-488-phalloidin (green). IF images were observed at 100×magnification. Arrows indicate N-WASP, Arp3 recruitment and F-actin tail formation. Enlargements of relevant region shown for clarity. Strains were assessed in two independent experiments. Scale bar = 10 µm. (C) Quantification of N-WASP/Arp3 recruitment, and (D) N-WASP/F-actin tail or capping formation, by intracellular *S. flexneri ΔicsA ΔrmlD* strains expressing IcsA::BIO only or co-expressing IcsA::BIO V382R (N-WASP IR II) and IcsA::BIO Y716G D717G (N-WASP IR III). Bacteria that either recruited both N-WASP and Arp3 (C), or recruited N-WASP and formed F-actin comet tail/capping (D), were scored from infected HeLa cells (*n* = 20 HeLa cells; ∼250–350 bacteria). Data are represented as percentage of N-WASP/Arp3 recruitment ± SEM of two independent experiments (C); and, as percentage of N-WASP recruitment/F-actin tail or capping formation ± SEM of two independent experiments (D). **, 0.001<*P*<0.01; ***, *P*<0.001 (determined by Student’s unpaired one-tailed *t* test). IR II & III = Interacting region II and III (*S. flexneri* co-expressing IcsA::BIO V382R and IcsA::BIO Y716G D717G).

To investigate if the N-WASP recruited by *S. flexneri* R-LPS co-expressing IcsA::BIO V382R and IcsA::BIO Y716G D717G (IR II+III) (MYRM697) was activated, Arp2/3 complex recruitment and F-actin comet tail formation were examined by IF microscopy with anti-Arp3 monoclonal antibody and Alexa Fluor 488-phalloidin staining, respectively. Arp3 recruitment was significantly detected at one pole of the bacterium which co-localised with N-WASP (Fig. 4A(b)) and F-actin comet tail/capping was observed (Fig. 4B(b)). The data indicate that the complementation of N-WASP interacting regions II and III allowed *S. flexneri* MYRM697 to activate N-WASP, recruit Arp2/3 complex and form F-actin comet tails. However, MYRM697 displayed reduced N-WASP recruitment frequency and F-actin comet tail/capping formation compared to MYRM704 (expressing the functionally equivalent IcsA::BIO). We have quantitated the recruitment of N-WASP, Arp3 and F-actin comet tail/capping formation by scoring 20 infected HeLa cells (∼250–350 bacteria) when both N-WASP/Arp3 and N-WASP/F-actin tail/capping were observed respectively, in two independent experiments. MYRM697 which co-expressed IcsA::BIO V382R and IcsA::BIO Y716G D717G had an approximately 40–45% reduction in N-WASP (P***<0.001) and Arp3 recruitment (0.001<P**<0.01) (Fig. 4C), as well as F-actin comet tail/capping formation (P***<0.001) (Fig. 4D), compared to MYRM704, the positive control strain. Nevertheless, the data shows significant co-operative interaction between N-WASP IRs on different IcsA molecules in N-WASP activation. However, we do not rule out that some degree of co-operativity between N-WASP IRs within the same IcsA molecule may also be occurring in this strain.

## Discussion

IcsA (VirG) is one of the key virulence proteins of *S. flexneri*, and is able to initiate F-actin comet tail formation through activation of the host N-WASP, leading to bacterial ABM and spreading within the host colonic epithelium. Although N-WASP is known to interact with IcsA and several IcsA regions have been reported to be involved in N-WASP recruitment [20], specific N-WASP interacting residues have yet to be identified. The aim of this study was to determine the N-WASP binding/interaction site(s) on IcsA (N-WASP interacting regions II and III) based on previously identified linker insertion mutations [20]. However, 5 aa linker insertion mutations may have caused local disruption to the IcsA secondary structure, complicating the identification of important amino acids residues involved in N-WASP interaction. Hence, site-directed mutagenesis of specific residues at these sites was undertaken in this study.

Specific amino acids within the N-WASP IR II were randomly mutated (aa 330–331 and aa 381–382, respectively). Screening of the resultant IcsA mutants suggested that for efficient N-WASP recruitment, a polar-polar combination is required for residues 330–331, while a non-polar amino acid is required for residue 382 (Tables 2 and 3). Hence, site-directed mutagenesis was subsequently performed on pIcsA::BIO to mutate residues G331 and V382 into non-polar tryptophan (W) and polar arginine (R), respectively. *S. flexneri* Δ*icsA* expressing IcsA::BIO G331W was capable of recruiting N-WASP and forming F-actin comet tail/capping but not as frequently as the positive control strain (IcsA::BIO). As a result, a reduction in plaque size (∼50%) was observed for IcsA::BIO G331W. In comparison, a single amino acid change at position 330 into a proline which is a non-polar amino acid (pMYRM539, T330P), abolished N-WASP recruitment. Notably, pMYRM543 (T330G G331P) which possesses proline at position 331 was also defective in N-WASP recruitment. Although both pMYRM543 (IcsA::BIO T330G G331P) and IcsA::BIO G331W mutants have the polar and non-polar amino acids combination, a contradictory effect on function was obtained. Taken together, the results obtained suggest that the proline substitution at either position disrupts local secondary structure of IcsA and prevents N-WASP binding. Nevertheless, the results suggest that N-WASP interaction at this site does not rely on amino acid polarity, and the polar-polar combination is not essential. Indeed, the data suggest that the presence of specific amino acid residues at positions 330–331 is required for efficient N-WASP interaction and recruitment.

Consistent with the IcsA::BIO T381*V382* mutants that also have residue 382 substituted with polar arginine (R) [pMYRM544 and pMYRM549] (Table 3), *S. flexneri* expressing the IcsA::BIO V382R mutant was incapable of recruiting N-WASP and forming plaques. In contrast, mutants T381M **V382L** [pMYRM547] and T381K **V382M** [pMYRM548] (Table 3), which possess a non-polar amino acid at residue 382 **(in bold)**, were able to recruit N-WASP and form plaques when expressed in *S. flexneri*, regardless of the amino acid substitution at residue 381. The data suggest that a non-polar residue is essential at position 382 and plays an important role in N-WASP recruitment. Collectively, site-directed mutagenesis within the N-WASP IR II revealed new N-WASP binding/interaction sites, and we have assigned them as N-WASP IR 2a (aa 330–331) and N-WASP IR 2b (aa 382), respectively.

We have previously shown that IcsA_i716_ which had a 5 aa insertion mutation within the AC region (N-WASP IR III) was defective in N-WASP recruitment [28]. The predicted location for residues Y716-D717 (forming part of the Aro-X-Aro motif) is at the beginning of the first anti-parallel β-strand that appears to be exposed to the external medium (Fig. S3), suggesting that this motif could be involved in protein binding. Hence, IcsA::BIO Y716*D717*, IcsA::BIO Y716F, IcsA::BIO Y716G and IcsA::BIO D717G mutants were created, expressed in both *S. flexneri* S-LPS and R-LPS backgrounds, and investigated for N-WASP recruitment. The IcsA::BIO Y716*D717* mutant protein production was markedly reduced in the S-LPS background but restored to a WT comparable levels in the R-LPS background (Fig. 2A), which is consistent with the IcsA_i716_ mutant phenotype [28]. In spite of the WT comparable levels of IcsA::BIO Y716*D717* mutant expression on the R-LPS bacterial surface, N-WASP recruitment was not observed (Table 4). The data suggested that this Aro-X-Aro motif is an N-WASP binding/interaction site, in this region of the protein.

Our results showed that IcsA::BIO Y716F (conserved mutation) and IcsA::BIO Y716G (non-conserved mutation) were functional in both S-LPS and R-LPS backgrounds, except that only F-actin capping was observed for Y716G in the S-LPS background. This may be due to the lower levels of IcsA::BIO Y716G expression in the S-LPS background which affected N-WASP recruitment efficiency and/or activation, hence, resulting in F-actin capping formation only. Consistent with these results, *S. flexneri* S-LPS expressing IcsA::BIO Y716F formed plaques that were comparable with WT while IcsA::BIO Y716G formed smaller plaques (Fig. 3B), reflecting the slightly reduced N-WASP recruitment efficiency and F-actin capping formation by IcsA::BIO Y716G. Together, the data suggest that the aromatic property of residue 716 is not essential for N-WASP recruitment.

IcsA::BIO D717G did not recruit N-WASP in the S-LPS background (Fig. 3A(g)), which is due to the extremely low IcsA production (Fig. 2C). Consequently, plaques were not observed. In contrast, the restoration of IcsA::BIO D717G expression in the R-LPS strain (Fig. 2C) did not lead to WT comparable N-WASP recruitment efficiency (Fig. 3A(h); Table 4), implying that D717 is involved in N-WASP binding/interaction. Collectively, the data obtained suggest that D717 plays a more important role than Y716 in N-WASP recruitment. Nevertheless, both residues Y716-D717 are important for N-WASP recruitment as mutation of both residues (IcsA::BIO Y716*D717* mutants) abolished N-WASP recruitment completely (Table 4).

In terms of IcsA biogenesis, we showed that the 716 aromatic residue is important for IcsA production but this was largely independent of LPS structure because the non-conservative mutation resulted in reduced IcsA::BIO Y716G protein production in both S-LPS and R-LPS backgrounds (Fig. 2B). This implies that the aromatic property of residue 716 is important for protein folding/stability. In contrast, D717 is a critical residue within the AC region for IcsA biogenesis but in a LPS structure dependent manner, as the IcsA::BIO D717G mutant had markedly reduced IcsA mutant production in the S-LPS background but was mostly restored in the R-LPS background (Fig. 2C, lanes 2 and 5). These results are consistent with our findings regarding the IcsA_i_ AC mutant production [28].

Previous investigations on IcsA regions involved in N-WASP recruitment have been studied on individual N-WASP interacting regions (based on deletion and linker insertion mutations) [20], [24], [26] and the identification of multiple N-WASP interacting regions on IcsA led us to investigate the mode of action of IcsA in recruiting and activating N-WASP. IcsA is part of the SAATs and has recently been shown to self-associate and oligomerise at the OM [18], [19]. On the other hand, allosterically activated N-WASP is able to dimerise/oligomerise and recruit the Arp2/3 complex [38], [39]. May *et al.* [19] also suggested that self-association of IcsA may play a role in N-WASP clustering, as *S. flexneri* S-LPS co-expressing IcsA_WT_ and IcsA_i563_ or IcsA_i677_ displayed reduced efficiency in ABM. We hypothesised that IcsA functions as a complex on the bacterial surface and different regions of IcsA (N-WASP IR I, II and III) are involved in interacting with N-WASP. To investigate our hypothesis, we co-expressed two IcsA mutant proteins in both S-LPS and R-LPS backgrounds in different combinations, to complement one mutated N-WASP IR with another copy of IcsA that had mutation at another N-WASP IR (Tables 5 and 6). Similar IcsA mutant production was obtained when the IcsA mutants were individually expressed by either pBR322 or pSU23 in S-LPS and R-LPS *S. flexneri*. Hence, the expression levels of co-expressed IcsA proteins (either with functional IcsA or IcsA mutant) were expected to be similar as the compatible intermediate copy plasmids (pBR322 and pSU23) were used. In addition, May *et al*. [19] showed that the co-expression of functional IcsA with IcsA_i_ mutant did not affect the polar distribution and the amount of functional protein at the bacterial surface.

Our findings showed that none of the IcsA mutants when co-expressed recruited N-WASP in the S-LPS background, except when co-expressed with IcsA_WT_ or the functionally equivalent IcsA::BIO (Table 5). Interestingly, when IcsA with mutations within N-WASP IR II and III (IcsA::BIO V382R and IcsA::BIO Y716G D717G) were co-expressed in the R-LPS background, N-WASP was activated, resulting in Arp2/3 complex recruitment and formation of F-actin comet tail/capping, albeit at a frequency less than the control (∼40–45% reduction) (Fig. 4). Co-expression of these IcsA mutant proteins in the S-LPS background did not show N-WASP recruitment which was likely due to the very low IcsA::BIO Y716G D717G production. IcsA::BIO V382R alone was not able to recruit N-WASP. Interestingly, this suggests that the AC region possessed by IcsA::BIO V382R does not seem to rescue the related expression defect of IcsA::BIO Y716G D717G. This is in contrast with the ability of the co-expressed AC region to rescue an AC region mutant in other systems (e.g. PrtS and BrkA ATs) [40], [41].

Studies on other autotransporter AC regions (e.g. PrtS and BrkA) were performed on *E. coli* which is a R-LPS strain, and the additional AC region was provided *in trans* and located at the OM [40], [41]. Our system is different from those previously reported whereby two full length mutated IcsA proteins were co-expressed in both S-LPS and R-LPS *S. flexneri*, and the AC IcsA mutant (e.g. IcsA::BIO Y716G D717G) production was severely affected in the S-LPS background. We have previously proposed that AC IcsA mutant could be degraded in the periplasm prior to translocation across the OM [28]. Hence, the failure to complement N-WASP recruitment by co-expressing IcsA::BIO V382R and IcsA::BIO Y716G D717G in the S-LPS background, could be due to the absence of IcsA::BIO Y716G D717G at the OM, as a result of degradation in the periplasm, supporting our previous hypothesis. Alternatively, LPS O-antigen could be masking the function of co-expressed IcsA::BIO V382R and IcsA::BIO Y716G D717G. However, it is unclear if IcsA::BIO Y716G D717G was co-expressed at high levels on the S-LPS *S. flexneri* surface. IcsA proteins with different tags could be co-expressed in S-LPS *S. flexneri* in future to determine the IcsA::BIO Y716G D717G expression level. In addition, we have previously shown that the expression of individual AC IcsA mutants in both R-LPS *S. flexneri* and R-LPS *E. coli* was restored to WT comparable levels, which does not require complementation of an additional AC region to restore IcsA biogenesis [28]. Therefore, it is unclear whether the AC region provided by IcsA::BIO V382R (at the OM) is able to restore IcsA biogenesis/folding of an AC IcsA mutant, as reported in PrtS and BrkA [40], [41].

**Figure 5:**
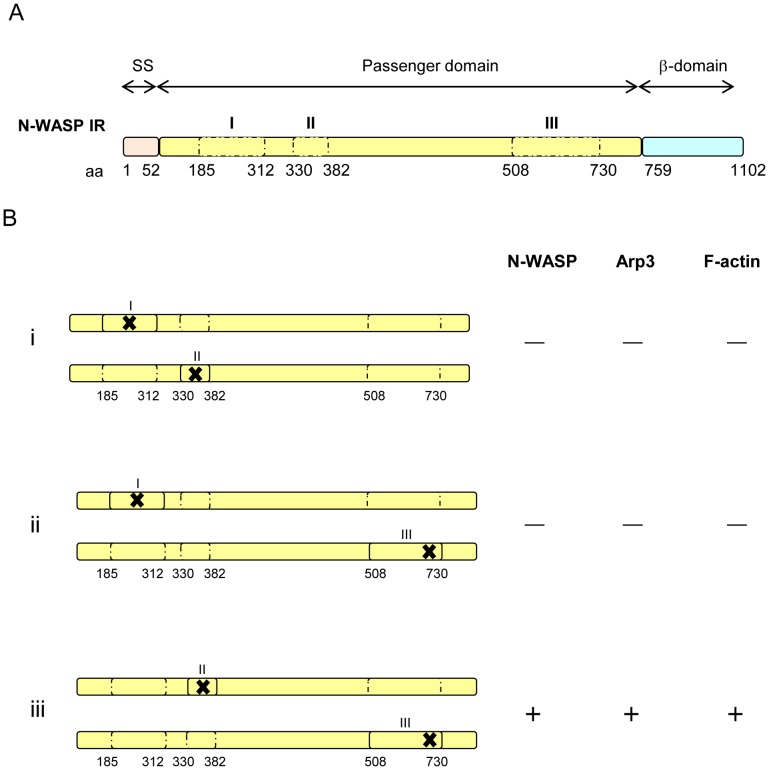
Schematic locations of the three N-WASP interacting regions with mutations in IcsA proteins. (A) Three major domains of IcsA. The N-WASP interacting regions (IR) are in dotted boxes and marked as I, II and III. (B) Three different combinations of IcsA mutants were co-expressed and the corresponding phenotypes in R-LPS *S. flexneri* are shown. (i) N-WASP IR I and II; (ii) N-WASP IR I and III; (iii) N-WASP IR II and III. “–” = N-WASP, Arp3 or F-actin comet tails not detected; “+” = N-WASP, Arp3 or F-actin comet tail/capping detected. The “✗” symbol indicates the presence of mutation within the N-WASP interacting region.

The N-WASP interacting region complementation data (Table 6; Fig. 4) suggest that IcsA functions as a complex (at least as a dimer), with all three N-WASP interacting regions required for efficient N-WASP recruitment. Furthermore, N-WASP IR II and III on different IcsA molecules interact co-operatively with N-WASP to promote its activation. However, we do not rule out that N-WASP binds to all three N-WASP IRs of one IcsA_WT_ molecule. The function of N-WASP IR I (contains the GRRs) cannot be complemented by another copy of IcsA with mutation at another N-WASP IR (Fig. 5). However, *S. flexneri* co-expressing both IcsA_i248_ (N-WASP IR I) and IcsA_WT_ (or the functionally equivalent IcsA::BIO) was capable of recruiting N-WASP, suggesting that despite mutation in N-WASP IR I, the presence of another copy of functional IcsA that provides sufficient functional N-WASP IRs is able to complement the defect, and a negative dominance effect was not detected.

Taken together, we have identified new amino acids within N-WASP IR II and III that are involved in N-WASP recruitment. In particular, the conserved Aro-X-Aro motif (aa 716–718, N-WASP IR III) that is located within the AC region could be a potential drug target site, by utilising the recently crystallised IcsA AC region structure. Furthermore, our N-WASP IR complementation data strongly suggest that IcsA functions as a multimer. We have shown for the first time that all three N-WASP IRs are involved in N-WASP recruitment and activation. Our findings have shed light on N-WASP activation by *S. flexneri* IcsA protein where the nature of their interactions is poorly understood. However, it is still unclear which region of N-WASP (WHI domain, CRIB domain or both) [26], [42] is involved in binding to the IcsA protein and whether N-WASP binds to three different sites of IcsA as a single molecule or as a complex. It is also unclear whether the binding of N-WASP to multiple sites of IcsA is a pre-requisite or if it merely enhances the allosteric activation of N-WASP and/or N-WASP oligomerisation which in turn activates the Arp2/3 complex. These issues warrant further investigations that would reveal the mystery of the complex interaction between the *S. flexneri* virulence IcsA protein and the host actin cytoskeleton regulatory N-WASP protein.

## Supporting Information

Figure S1 **Plaque formation by** ***S. flexneri*** **Δ** ***icsA*** expressing IcsA::BIO T330*G331* and IcsA::BIO T381*V382* mutants. Confluent HeLa cell monolayers were infected with mid-exponential phase *S. flexneri* strains for 2 h, and plaques were observed 48 h post-infection as detailed in Materials and Methods. 30 plaques were measured from each experiment. Data are represented as mean ± SEM of three independent experiments. **, 0.001<*P*<0.01; ***, *P*<0.001 (determined by Student’s unpaired one-tailed *t* test).
(TIF)

Figure S2 **Detection of surface IcsA::BIO, IcsA::BIO G331W or IcsA::BIO V382R proteins expressed by S-LPS** ***S. flexneri*** **.** Mid-exponential phase *S. flexneri* Δ*icsA* strains expressing either IcsA::BIO, IcsA::BIO G331W, IcsA::BIO V382R or an empty vector alone, were formalin fixed and labelled with rabbit polyclonal anti-IcsA antibody and Alexa 488-conjugated goat anti-rabbit secondary antibody. Insert shows an enlargement for greater clarity. IF images were observed at 100×magnification. Scale bar = 10 µm.
(TIF)

Figure S3 **Locations of Y716, D717 and Y718 residues mapped on the crystal structure of the IcsA AC region.** Protein Data Bank accession no. 3ML3. Residue Y716 is illustrated in pink, residue D717 is yellow and residue Y718 is blue. Side chains of each residue, as well as the amino (N) and carboxyl (C) termini are shown.
(TIF)

Figure S4 **Detection of surface IcsA::BIO Y716 or D717 proteins expressed by S-LPS and R-LPS** ***S. flexneri*** **.** Mid-exponential phase *S. flexneri* Δ*icsA* (S-LPS) or *S. flexneri* Δ*icsA* Δ*rmlD* (R-LPS) strains expressing either IcsA::BIO, IcsA::BIO Y716F, IcsA::BIO Y716G, IcsA::BIO D717G or an empty vector, were formalin fixed and labelled with anti-IcsA antibody and Alexa 488-conjugated goat anti-rabbit secondary antibody. Insert shows an enlargement for greater clarity. IF images were observed at 100×magnification. Scale bar = 10 µm.
(TIF)

Figure S5 **Detection of IcsA proteins encoded by pSU23-based plasmids in S-LPS and R-LPS** ***S. flexneri*** **.** (A) Whole cell lysates from mid-exponential phase *S. flexneri ΔicsA* (S-LPS) or *S. flexneri* Δ*icsA* Δ*rmlD* (R-LPS) expressing IcsA_WT_, IcsA_i248_, IcsA::BIO IcsA::BIO Y716G D717G or an empty vector were prepared and analysed by Western immunoblotting using anti-IcsA antibody. Strain names are shown above each lane. The 120 kDa band corresponds to the full length IcsA; the 85 kDa band corresponds to the cleaved form (IcsA’). S = S-LPS; R = R-LPS. (B) Mid-exponential phase *S. flexneri* Δ*icsA* (S-LPS) or *S. flexneri* Δ*icsA* Δ*rmlD* (R-LPS) strains expressing either IcsA, IcsA::BIO, IcsA_i248_, IcsA::BIO Y716G D717G or an empty vector, were formalin fixed and labelled with rabbit polyclonal anti-IcsA antibody and Alexa 488-conjugated goat anti-rabbit secondary antibody. Insert shows an enlargement for greater clarity. IF images were observed at 100×magnification. Scale bar = 10 µm.
(TIF)

Table S1 Oligonucleotides used in this study.
(TIF)

Table S2 IcsA production and N-WASP recruitment by S. flexneri S-LPS and R-LPS straisn expressing various IcsA proteins.
(TIF)

## References

[1] Philpott DJ, Edgeworth JD, Sansonetti PJ (2000) The pathogenesis of *Shigella flexneri* infection: lessons from in vitro and in vivo studies. Philos Trans R Soc Lond B Biol Sci 355: 575–586. 10874731 10.1098/rstb.2000.0599 PMC1692768

[2] Bernardini ML, Mounier J, d’Hauteville H, Coquis-Rondon M, Sansonetti PJ (1989) Identification of *icsA*, a plasmid locus of *Shigella flexneri* that governs bacterial intra- and intercellular spread through interaction with F-actin. Proc Natl Acad Sci U S A 86: 3867–3871. 2542950 10.1073/pnas.86.10.3867 PMC287242

[3] Suzuki T, Sasakawa C (2001) Molecular basis of the intracellular spreading of *Shigella* . Infect Immun 69: 5959–5966. 11553531 10.1128/IAI.69.10.5959-5966.2001 PMC98722

[4] Lett MC, Sasakawa C, Okada N, Sakai T, Makino S, et al (1989) *virG*, a plasmid-coded virulence gene of *Shigella flexneri*: identification of the virG protein and determination of the complete coding sequence. J Bacteriol 171: 353–359. 2644195 10.1128/jb.171.1.353-359.1989 PMC209595

[5] Makino S, Sasakawa C, Kamata K, Kurata T, Yoshikawa M (1986) A genetic determinant required for continuous reinfection of adjacent cells on large plasmid in *S. flexneri* 2a. Cell 46: 551–555. 3524856 10.1016/0092-8674(86)90880-9

[6] Sansonetti PJ, Arondel J, Fontaine A, d’Hauteville H, Bernardini ML (1991) OmpB (osmo-regulation) and *icsA* (cell-to-cell spread) mutants of *Shigella flexneri*: vaccine candidates and probes to study the pathogenesis of shigellosis. Vaccine 9: 416–422. 1887672 10.1016/0264-410x(91)90128-s

[7] Cossart P (2000) Actin-based motility of pathogens: the Arp2/3 complex is a central player. Cell Microbiol 2: 195–205. 11207576 10.1046/j.1462-5822.2000.00053.x

[8] Goldberg MB (2001) Actin-based motility of intracellular microbial pathogens. Microbiol Mol Biol Rev 65: 595–626, table of contents. 10.1128/MMBR.65.4.595-626.2001 PMC99042 11729265

[9] Pantaloni D, Le Clainche C, Carlier MF (2001) Mechanism of actin-based motility. Science 292: 1502–1506. 11379633 10.1126/science.1059975

[10] Pallen MJ, Chaudhuri RR, Henderson IR (2003) Genomic analysis of secretion systems. Curr Opin Microbiol 6: 519–527. 14572546 10.1016/j.mib.2003.09.005

[11] Henderson IR, Navarro-Garcia F, Desvaux M, Fernandez RC, Ala’Aldeen D (2004) Type V protein secretion pathway: the autotransporter story. Microbiol Mol Biol Rev 68: 692–744. 15590781 10.1128/MMBR.68.4.692-744.2004 PMC539010

[12] Jain S, Goldberg MB (2007) Requirement for YaeT in the outer membrane assembly of autotransporter proteins. J Bacteriol 189: 5393–5398. 17513479 10.1128/JB.00228-07 PMC1951886

[13] Suzuki T, Lett MC, Sasakawa C (1995) Extracellular transport of VirG protein in *Shigella* . J Biol Chem 270: 30874–30880. 8537341 10.1074/jbc.270.52.30874

[14] Brandon LD, Goehring N, Janakiraman A, Yan AW, Wu T, et al (2003) IcsA, a polarly localized autotransporter with an atypical signal peptide, uses the Sec apparatus for secretion, although the Sec apparatus is circumferentially distributed. Mol Microbiol 50: 45–60. 14507362 10.1046/j.1365-2958.2003.03674.x

[15] Peterson JH, Tian P, Ieva R, Dautin N, Bernstein HD (2010) Secretion of a bacterial virulence factor is driven by the folding of a C-terminal segment. Proc Natl Acad Sci U S A 107: 17739–17744. 20876094 10.1073/pnas.1009491107 PMC2955144

[16] Wells TJ, Totsika M, Schembri MA (2010) Autotransporters of *Escherichia coli*: a sequence-based characterization. Microbiology 156: 2459–2469. 20447993 10.1099/mic.0.039024-0

[17] Klemm P, Vejborg RM, Sherlock O (2006) Self-associating autotransporters, SAATs: functional and structural similarities. Int J Med Microbiol 296: 187–195. 16600681 10.1016/j.ijmm.2005.10.002

[18] Meng G, Spahich N, Kenjale R, Waksman G, St Geme JW 3rd (2011) Crystal structure of the *Haemophilus influenzae* Hap adhesin reveals an intercellular oligomerization mechanism for bacterial aggregation. EMBO J 30: 3864–3874. 21841773 10.1038/emboj.2011.279 PMC3173798

[19] May KL, Grabowicz M, Polyak SW, Morona R (2012) Self-association of the *Shigella flexneri* IcsA autotransporter protein. Microbiology 158: 1874–1883. 22516224 10.1099/mic.0.056465-0

[20] May KL, Morona R (2008) Mutagenesis of the *Shigella flexneri* autotransporter IcsA reveals novel functional regions involved in IcsA biogenesis and recruitment of host neural Wiscott-Aldrich syndrome protein. J Bacteriol 190: 4666–4676. 18456802 10.1128/JB.00093-08 PMC2446779

[21] Kuhnel K, Diezmann D (2011) Crystal structure of the autochaperone region from the *Shigella flexneri* autotransporter IcsA. J Bacteriol 193: 2042–2045. 21335457 10.1128/JB.00790-10 PMC3133035

[22] Yarar D, To W, Abo A, Welch MD (1999) The Wiskott-Aldrich syndrome protein directs actin-based motility by stimulating actin nucleation with the Arp2/3 complex. Curr Biol 9: 555–558. 10339430 10.1016/s0960-9822(99)80243-7

[23] Snapper SB, Takeshima F, Anton I, Liu CH, Thomas SM, et al (2001) N-WASP deficiency reveals distinct pathways for cell surface projections and microbial actin-based motility. Nat Cell Biol 3: 897–904. 11584271 10.1038/ncb1001-897

[24] Suzuki T, Miki H, Takenawa T, Sasakawa C (1998) Neural Wiskott-Aldrich syndrome protein is implicated in the actin-based motility of *Shigella flexneri* . EMBO J 17: 2767–2776. 9582270 10.1093/emboj/17.10.2767 PMC1170617

[25] Miki H, Takenawa T (2003) Regulation of actin dynamics by WASP family proteins. J Biochem 134: 309–313. 14561714 10.1093/jb/mvg146

[26] Suzuki T, Mimuro H, Suetsugu S, Miki H, Takenawa T, et al (2002) Neural Wiskott-Aldrich syndrome protein (N-WASP) is the specific ligand for *Shigella* VirG among the WASP family and determines the host cell type allowing actin-based spreading. Cell Microbiol 4: 223–233. 11952639 10.1046/j.1462-5822.2002.00185.x

[27] Ogawa M, Yoshimori T, Suzuki T, Sagara H, Mizushima N, et al (2005) Escape of intracellular *Shigella* from autophagy. Science 307: 727–731. 15576571 10.1126/science.1106036

[28] Teh MY, Tran EN, Morona R (2012) Absence of O-antigen suppresses *Shigella flexneri* IcsA autochaperone region mutations. Microbiology 158: 2835–2850. 22936034 10.1099/mic.0.062471-0

[29] Morona R, Daniels C, Van Den Bosch L (2003) Genetic modulation of *Shigella flexneri* 2a lipopolysaccharide O antigen modal chain length reveals that it has been optimized for virulence. Microbiology 149: 925–939. 12686635 10.1099/mic.0.26141-0

[30] Baker SJ, Gunn JS, Morona R (1999) The *Salmonella typhi* melittin resistance gene *pqaB* affects intracellular growth in PMA-differentiated U937 cells, polymyxin B resistance and lipopolysaccharide. Microbiology 145 (Pt 2): 367–378. 10.1099/13500872-145-2-367 10075419

[31] Morona R, van den Bosch L, Manning PA (1995) Molecular, genetic, and topological characterization of O-antigen chain length regulation in *Shigella flexneri* . J Bacteriol 177: 1059–1068. 7532168 10.1128/jb.177.4.1059-1068.1995 PMC176702

[32] Van Den Bosch L, Manning PA, Morona R (1997) Regulation of O-antigen chain length is required for *Shigella flexneri* virulence. Mol Microbiol 23: 765–775. 9157247 10.1046/j.1365-2958.1997.2541625.x

[33] Lugtenberg B, Meijers J, Peters R, van der Hoek P, van Alphen L (1975) Electrophoretic resolution of the “major outer membrane protein” of *Escherichia coli* K12 into four bands. FEBS Lett 58: 254–258. 773686 10.1016/0014-5793(75)80272-9

[34] Cull MG, Schatz PJ (2000) Biotinylation of proteins in vivo and in vitro using small peptide tags. Methods in enzymology 326: 430–440. 11036656 10.1016/s0076-6879(00)26068-0

[35] Oaks EV, Wingfield ME, Formal SB (1985) Plaque formation by virulent *Shigella flexneri* . Infect Immun 48: 124–129. 3884506 10.1128/iai.48.1.124-129.1985 PMC261924

[36] Bitto E, McKay DB (2003) The periplasmic molecular chaperone protein SurA binds a peptide motif that is characteristic of integral outer membrane proteins. J Biol Chem 278: 49316–49322. 14506253 10.1074/jbc.M308853200

[37] Xu X, Wang S, Hu YX, McKay DB (2007) The periplasmic bacterial molecular chaperone SurA adapts its structure to bind peptides in different conformations to assert a sequence preference for aromatic residues. J Mol Biol 373: 367–381. 17825319 10.1016/j.jmb.2007.07.069 PMC2040117

[38] Padrick SB, Rosen MK (2010) Physical mechanisms of signal integration by WASP family proteins. Annu Rev Biochem 79: 707–735. 20533885 10.1146/annurev.biochem.77.060407.135452 PMC3017724

[39] Padrick SB, Cheng HC, Ismail AM, Panchal SC, Doolittle LK, et al (2008) Hierarchical regulation of WASP/WAVE proteins. Mol Cell 32: 426–438. 18995840 10.1016/j.molcel.2008.10.012 PMC2680354

[40] Ohnishi Y, Nishiyama M, Horinouchi S, Beppu T (1994) Involvement of the COOH-terminal pro-sequence of Serratia marcescens serine protease in the folding of the mature enzyme. J Biol Chem 269: 32800–32806. 7806503

[41] Oliver DC, Huang G, Nodel E, Pleasance S, Fernandez RC (2003) A conserved region within the *Bordetella pertussis* autotransporter BrkA is necessary for folding of its passenger domain. Mol Microbiol 47: 1367–1383. 12603741 10.1046/j.1365-2958.2003.03377.x

[42] Moreau V, Frischknecht F, Reckmann I, Vincentelli R, Rabut G, et al (2000) A complex of N-WASP and WIP integrates signalling cascades that lead to actin polymerization. Nat Cell Biol 2: 441–448. 10878810 10.1038/35017080

[43] Van Den Bosch L, Morona R (2003) The actin-based motility defect of a *Shigella flexneri rmlD* rough LPS mutant is not due to loss of IcsA polarity. Microb Pathog 35: 11–18. 12860454 10.1016/s0882-4010(03)00064-0

[44] Bolivar F, Rodriguez RL, Betlach MC, Boyer HW (1977) Construction and characterization of new cloning vehicles. I. Ampicillin-resistant derivatives of the plasmid pMB9. Gene 2: 75–93. 344136 10.1016/0378-1119(77)90074-9

[45] Bartolome B, Jubete Y, Martinez E, de la Cruz F (1991) Construction and properties of a family of pACYC184-derived cloning vectors compatible with pBR322 and its derivatives. Gene 102: 75–78. 1840539 10.1016/0378-1119(91)90541-i

