# Supplementary figures and images for: Identification of Shigella flexneri IcsA Residues Affecting Interaction with N-WASP, and Evidence for IcsA-IcsA Co-Operative Interaction
Source: PLoS One. 2013 Feb 6;8(2):e55152. doi: 10.1371/journal.pone.0055152 (PMC3566212; doi:10.1371/journal.pone.0055152)

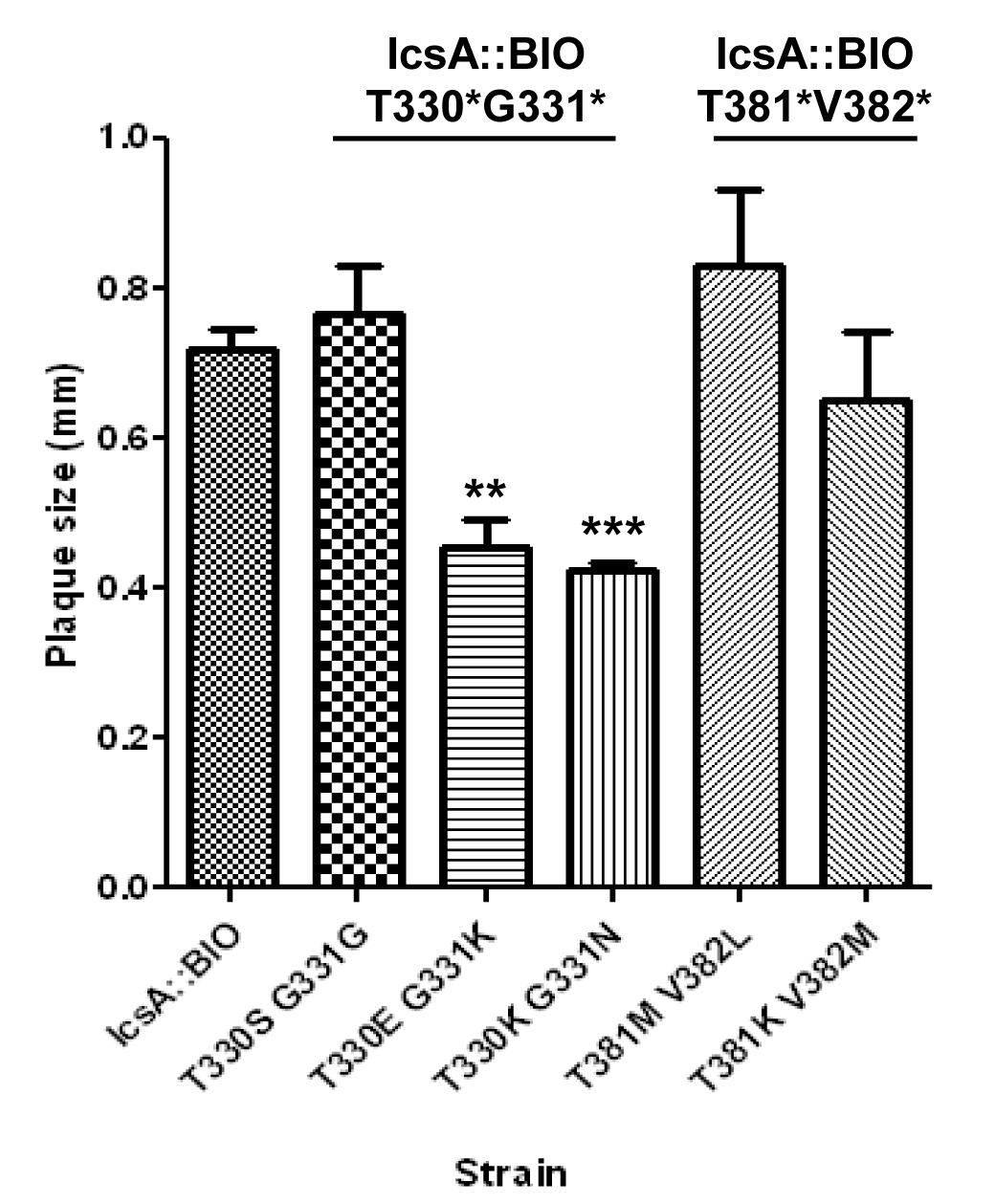

Supplement: Figure S1 — Plaque formation by S. flexneri Δ icsA expressing IcsA::BIO T330*G331* and IcsA::BIO T381*V382* mutants. Confluent HeLa cell monolayers were infected with mid-exponential phase S. flexneri strains for 2 h, and plaques were observed 48 h post-infection as detailed in Materials and Methods. 30 plaques were measured from each experiment. Data are represented as mean ± SEM of three independent experiments. **, 0.001<P<0.01; ***, P<0.001 (determined by Student’s unpaired one-tailed t test). (TIF) [file pone.0055152.s001.tif]

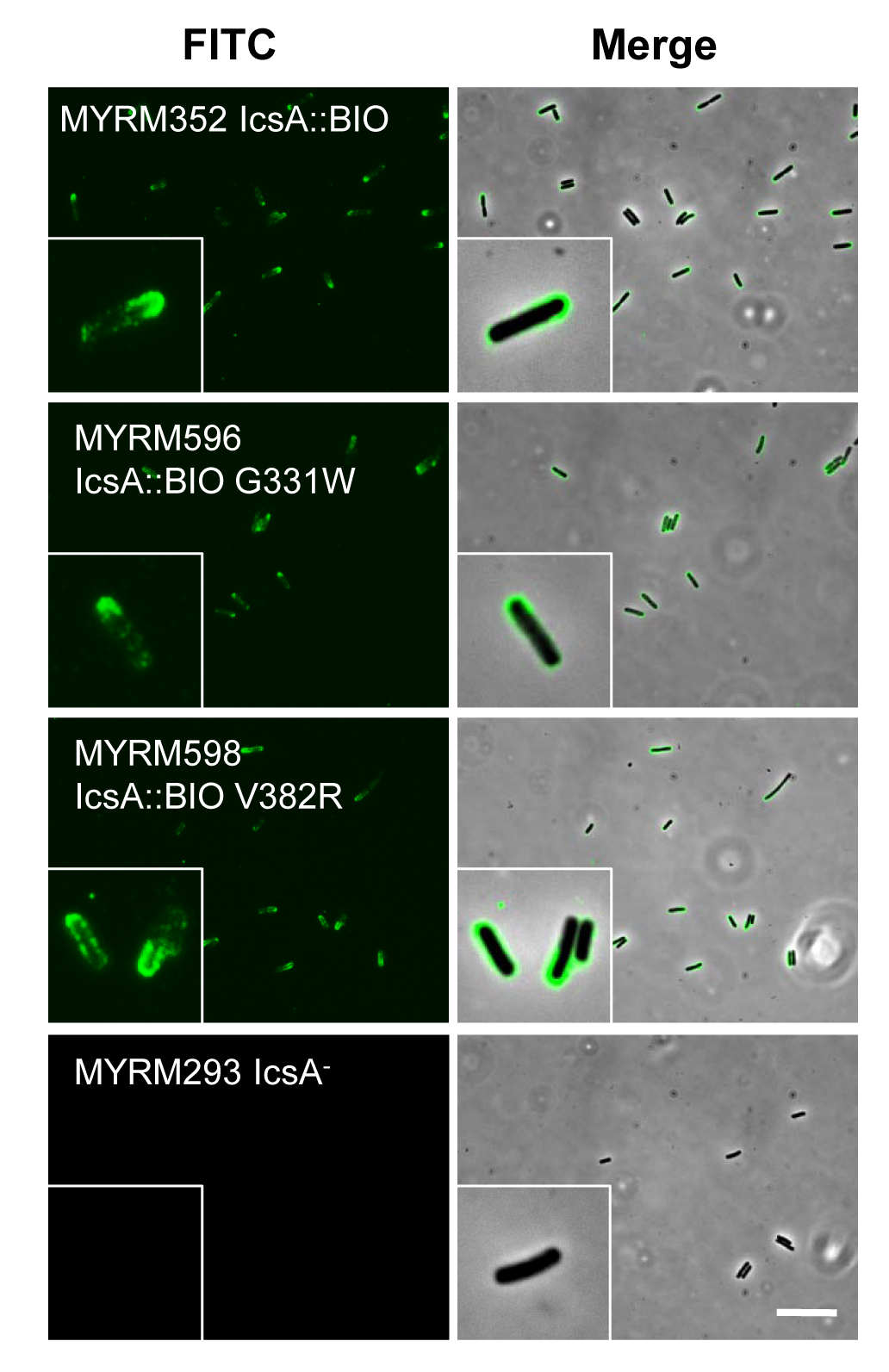

Supplement: Figure S2 — Detection of surface IcsA::BIO, IcsA::BIO G331W or IcsA::BIO V382R proteins expressed by S-LPS S. flexneri . Mid-exponential phase S. flexneri ΔicsA strains expressing either IcsA::BIO, IcsA::BIO G331W, IcsA::BIO V382R or an empty vector alone, were formalin fixed and labelled with rabbit polyclonal anti-IcsA antibody and Alexa 488-conjugated goat anti-rabbit secondary antibody. Insert shows an enlargement for greater clarity. IF images were observed at 100×magnification. Scale bar = 10 µm. (TIF) [file pone.0055152.s002.tif]

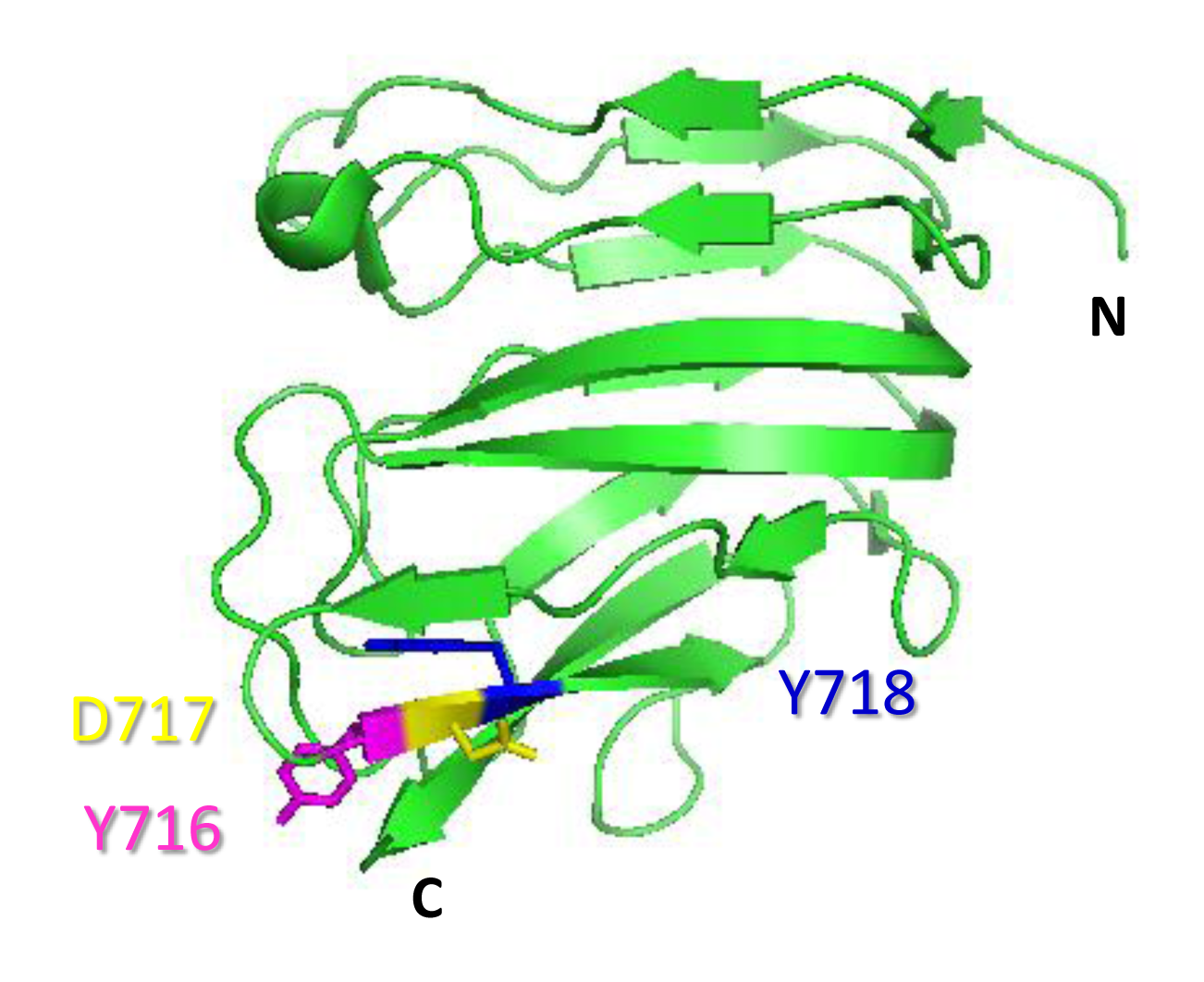

Supplement: Figure S3 — Locations of Y716, D717 and Y718 residues mapped on the crystal structure of the IcsA AC region. Protein Data Bank accession no. 3ML3. Residue Y716 is illustrated in pink, residue D717 is yellow and residue Y718 is blue. Side chains of each residue, as well as the amino (N) and carboxyl (C) termini are shown. (TIF) [file pone.0055152.s003.tif]

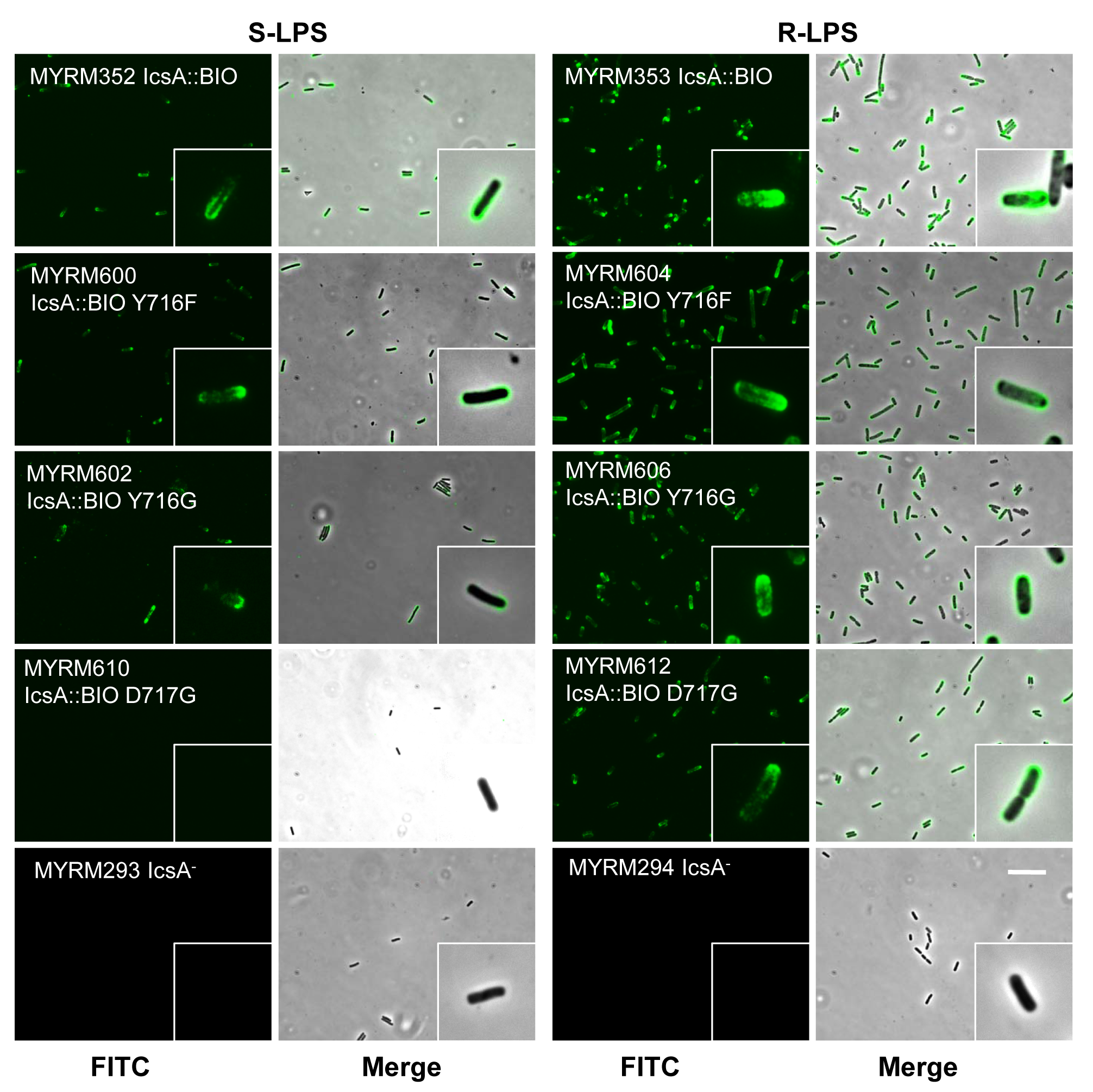

Supplement: Figure S4 — Detection of surface IcsA::BIO Y716 or D717 proteins expressed by S-LPS and R-LPS S. flexneri . Mid-exponential phase S. flexneri ΔicsA (S-LPS) or S. flexneri ΔicsA ΔrmlD (R-LPS) strains expressing either IcsA::BIO, IcsA::BIO Y716F, IcsA::BIO Y716G, IcsA::BIO D717G or an empty vector, were formalin fixed and labelled with anti-IcsA antibody and Alexa 488-conjugated goat anti-rabbit secondary antibody. Insert shows an enlargement for greater clarity. IF images were observed at 100×magnification. Scale bar = 10 µm. (TIF) [file pone.0055152.s004.tif]

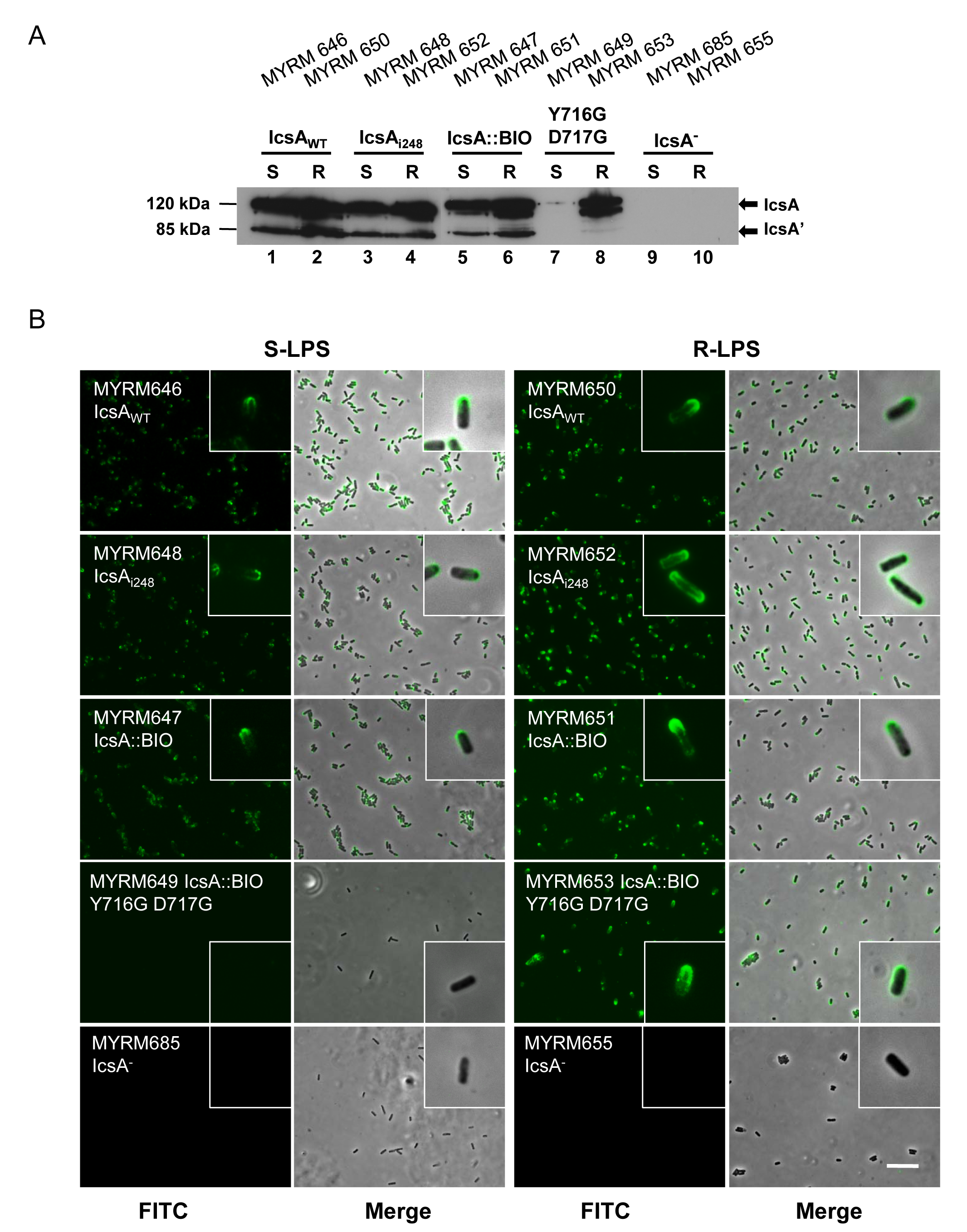

Supplement: Figure S5 — Detection of IcsA proteins encoded by pSU23-based plasmids in S-LPS and R-LPS S. flexneri . (A) Whole cell lysates from mid-exponential phase S. flexneri ΔicsA (S-LPS) or S. flexneri ΔicsA ΔrmlD (R-LPS) expressing IcsAWT, IcsAi248, IcsA::BIO IcsA::BIO Y716G D717G or an empty vector were prepared and analysed by Western immunoblotting using anti-IcsA antibody. Strain names are shown above each lane. The 120 kDa band corresponds to the full length IcsA; the 85 kDa band corresponds to the cleaved form (IcsA’). S = S-LPS; R = R-LPS. (B) Mid-exponential phase S. flexneri ΔicsA (S-LPS) or S. flexneri ΔicsA ΔrmlD (R-LPS) strains expressing either IcsA, IcsA::BIO, IcsAi248, IcsA::BIO Y716G D717G or an empty vector, were formalin fixed and labelled with rabbit polyclonal anti-IcsA antibody and Alexa 488-conjugated goat anti-rabbit secondary antibody. Insert shows an enlargement for greater clarity. IF images were observed at 100×magnification. Scale bar = 10 µm. (TIF) [file pone.0055152.s005.tif]

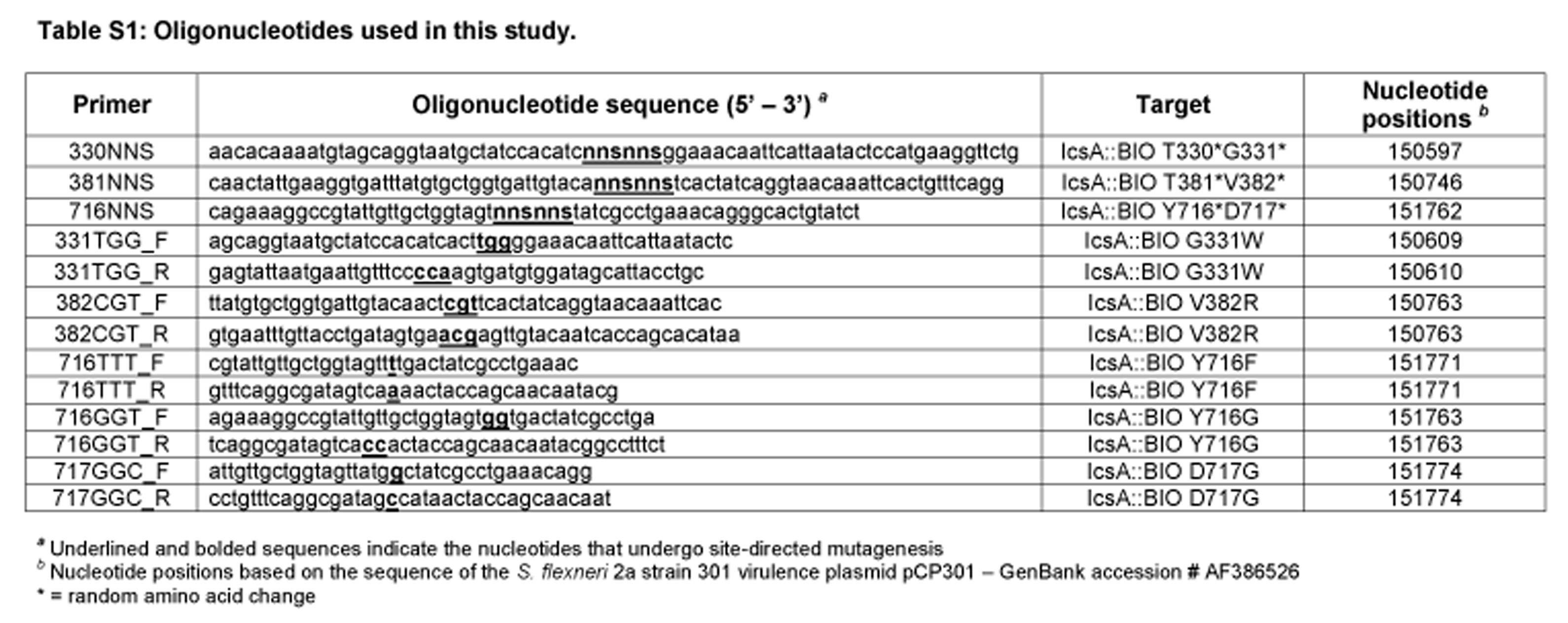

Supplement: Table S1 — Oligonucleotides used in this study. (TIF) [file pone.0055152.s006.tif]

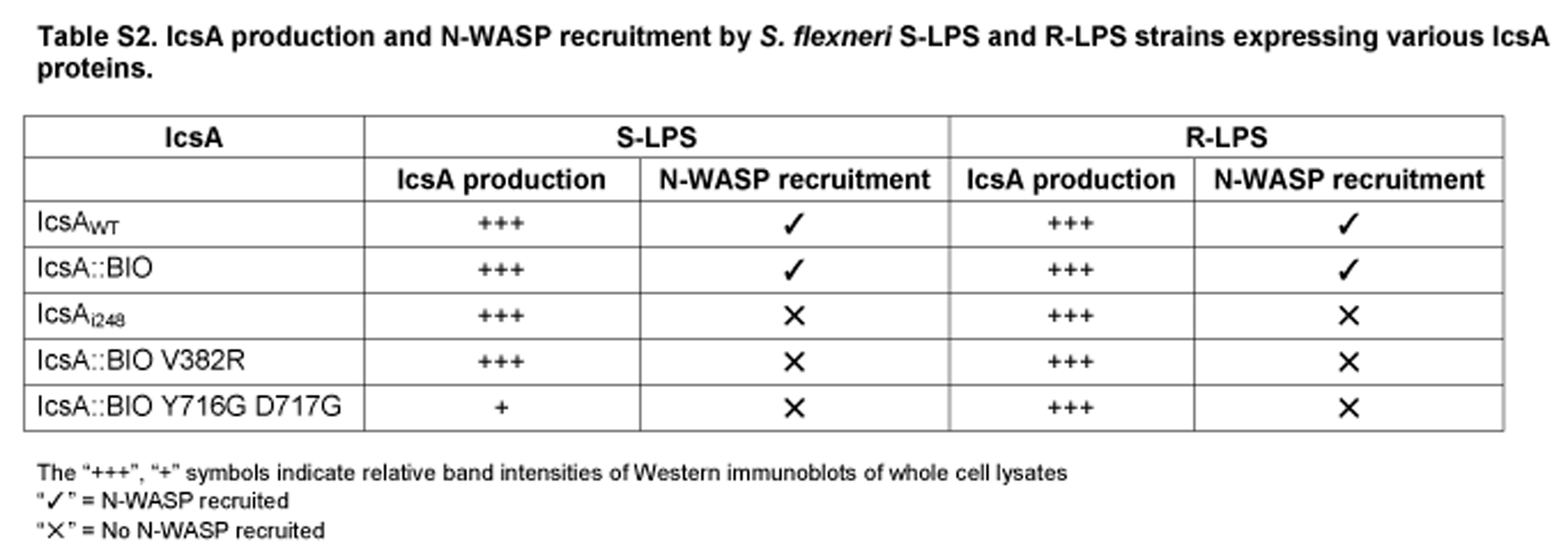

Supplement: Table S2 — IcsA production and N-WASP recruitment by S. flexneri S-LPS and R-LPS straisn expressing various IcsA proteins. (TIF) [file pone.0055152.s007.tif]
